# The pomegranate (*Punica granatum* L.) genome provides insights into fruit quality and ovule developmental biology

**DOI:** 10.1111/pbi.12875

**Published:** 2018-01-22

**Authors:** Zhaohe Yuan, Yanming Fang, Taikui Zhang, Zhangjun Fei, Fengming Han, Cuiyu Liu, Min Liu, Wei Xiao, Wenjing Zhang, Shan Wu, Mengwei Zhang, Youhui Ju, Huili Xu, He Dai, Yujun Liu, Yanhui Chen, Lili Wang, Jianqing Zhou, Dian Guan, Ming Yan, Yanhua Xia, Xianbin Huang, Dongyuan Liu, Hongmin Wei, Hongkun Zheng

**Affiliations:** ^1^ Co‐Innovation Center for Sustainable Forestry in Southern China Nanjing Forestry University Nanjing China; ^2^ College of Forestry Nanjing Forestry University Nanjing China; ^3^ College of Biology and the Environment Nanjing Forestry University Nanjing China; ^4^ Boyce Thompson Institute Cornell University Ithaca NY USA; ^5^ USDA Robert W. Holley Center for Agriculture and Health Ithaca NY USA; ^6^ Biomarker Technologies Corporation Beijing China; ^7^ College of Biological Sciences and Biotechnology Beijing Forestry University Beijing China; ^8^ College of Horticulture Henan Agricultural University Zhengzhou China

**Keywords:** *Punica granatum*, genome assembly, phylogenomic analysis, fruit quality development, ovule development

## Abstract

Pomegranate (*Punica granatum* L.) has an ancient cultivation history and has become an emerging profitable fruit crop due to its attractive features such as the bright red appearance and the high abundance of medicinally valuable ellagitannin‐based compounds in its peel and aril. However, the limited genomic resources have restricted further elucidation of genetics and evolution of these interesting traits. Here, we report a 274‐Mb high‐quality draft pomegranate genome sequence, which covers approximately 81.5% of the estimated 336‐Mb genome, consists of 2177 scaffolds with an N50 size of 1.7 Mb and contains 30 903 genes. Phylogenomic analysis supported that pomegranate belongs to the Lythraceae family rather than the monogeneric Punicaceae family, and comparative analyses showed that pomegranate and *Eucalyptus grandis* share the paleotetraploidy event. Integrated genomic and transcriptomic analyses provided insights into the molecular mechanisms underlying the biosynthesis of ellagitannin‐based compounds, the colour formation in both peels and arils during pomegranate fruit development, and the unique ovule development processes that are characteristic of pomegranate. This genome sequence provides an important resource to expand our understanding of some unique biological processes and to facilitate both comparative biology studies and crop breeding.

## Introduction

Pomegranate (*Punica granatum* L.), native to central Asia, is an ancient medicinal fruit crop grown worldwide (Holland *et al*., 2009) that has considerable economic value. Although the genus *Punica* was previously placed in its own monogeneric family (Punicaceae), recent morphological (Graham and Graham, 2014) and molecular (Berger *et al*., 2016) evidence, as well as the new classification in the APG IV system (Byng *et al*., 2016), suggests that it is instead a member of Lythraceae.

Compared to other fruit crops, such as orange (*Citrus sinensis*), apple (*Malus domestica*), grape (*Vitis vinifera*) and kiwifruit (*Actinidia chinensis*), pomegranate has higher levels of antioxidants (~11.33 mmol/100 g) (Halvorsen *et al*., 2002), which are potentially beneficial in preventing cardiovascular disease, diabetes and prostate cancer (Johanningsmeier and Harris, 2011). Consequently, pomegranate is referred to as a ‘super fruit’ (Teixeira da Silva *et al*., 2013) and the planted acreages and fruit production of pomegranate have increased substantially over the past decade (Yi *et al*., 2016).

Apart from it commercial importance, pomegranate has become an attractive system for studying several valuable biological features, such as high antioxidant activity in the fruit, colour formation in the fruit peel and aril (the edible part of the pomegranate fruit), and poly‐caryopsis as a valuable trait for crop production and ovule developmental biology (Supporting Information). High contents of punicalagins and other ellagitannin‐based compounds mainly contribute to the high antioxidant activity in pomegranate fruit (Johanningsmeier and Harris, 2011). Genetic and physiological studies show that the UDP‐glucose:gallate glucosyltransferase (UGT) gene plays a key role in the ellagitannin biosynthesis, catalysing gallic acid to β‐glucogallin in pomegranate (Ono *et al*., 2016). However, to date very limited information is available in understanding the production of punicalagins in pomegranate. Peel and aril colour, as a consequence of the anthocyanin accumulation, is a critical trait in determining pomegranate fruit commodity value and quality. Although previous transcriptomic studies have deciphered a peel‐specific anthocyanin biosynthesis pathway (Ono *et al*., 2011) and a regulatory network (Ben‐Simhon *et al*., 2011), little has been reported regarding the pathway in aril, and the large omics view on fruit colour development. Pomegranate possesses arils and more than one hundred ovules grow in one pomegranate ovary (Teixeira da Silva *et al*., 2013), making pomegranate an ideal system for studying ovule developmental biology. Despite of a detailed knowledge base of ovule developmental biology based on model species like *Arabidopsis* (Colombo *et al*., 2008), there have been very few related studies in pomegranate.

Genomic resources, which have great values for both basic research and crop improvement, are currently very limited for pomegranate. We have therefore sequenced and assembled the genome of *P. granatum* ‘Taishanhong’, a widely grown cultivar in China that exhibits bright red fruit at the ripe stage. Genome and transcriptome analyses presented in this study provide insights into the pomegranate taxonomic status and evolution, as well as the molecular mechanisms underlying ellagitannin‐based compound metabolism, anthocyanin biosynthesis and ovule development.

## Results

### Genome assembly and annotation

We used the whole‐genome shotgun sequencing approach to generate ~67 Gb of high‐quality sequences (Table S1), representing approximately 200× coverage of the pomegranate genome, which has an estimated size of 336 Mb based on the K‐mer depth distribution analysis of the sequenced reads (Figure S1) and the flow cytometry analysis (Table S2). The final assembled sequence was 274 Mb, representing 81.5% of the pomegranate genome. The assembly consisted of 2177 scaffolds (≥1 kb) with an N50 of 1.7 Mb and 7088 contigs with an N50 of 97 kb (Table 1; Table S3). The GC content of the assembled pomegranate genome was 39.2%, similar to that of *Eucalyptus grandis*, the most closely related species to pomegranate with a sequenced genome (Myburg *et al*., 2014).

**Table 1:** Statistics of pomegranate genome assembly and annotation

Estimated genome size (Mb)	336
Total size of assembled scaffolds (Mb)	274
Number of scaffolds (≥1 kb)	2117
N50 scaffold length (Mb)	1.7
Longest scaffold (Mb)	7.6
Total size of assembled contigs (Mb)	269
Number of contigs (≥1 kb)	7088
N50 contig length (Kb)	97.0
Largest contig (Kb)	528.6
GC content (%)	39.2
Number of gene models	30 903
Mean transcript length (bp)	2332.8
Mean coding sequence length (bp)	1110.4
Mean number of exons per gene	4.52
Mean exon length (bp)	245.9
Mean intron length (bp)	347.6

We first assessed the quality of the assembled pomegranate genome using BUSCO (Simao *et al*., 2015), which revealed that 94.3% (1358 out of 1440) of the core eukaryotic genes were captured by the pomegranate genome assembly and that 91.6% (1319 of 1440) were complete. In addition, our assembled sequence covered >99% of the 2397 pomegranate expressed sequence tags (ESTs) downloaded from GenBank (Table S4). Finally, the assembled genome covered >94% of the unigenes assembled from our pomegranate RNA‐Seq data (Table S5). Taken together, these results indicate that the assembled pomegranate genome is of high quality.

We predicted a total of 30 903 protein‐coding genes in the pomegranate genome, with a mean coding sequence length of 1110 bp and 4.5 exons per gene (Table 1). Of these genes, 89% could be annotated using the GO (Ashburner *et al*., 2000), KEGG (Kanehisa and Goto, 2000), TrEMBL (Bairoch and Apweiler, 1997), COG (Tatusov *et al*., 2000), or the GenBank nr databases (Table S6). Conserved domains in 80% of the predicted proteins were identified by comparing them against the InterPro database (Mitchell *et al*., 2014). In addition to the protein‐coding genes, 601 miRNA, 54 rRNA and 144 tRNA genes were also identified in the pomegranate genome (Table S7).

### Repetitive sequence evolution

Repetitive sequences generally constitute a large portion of a plant genome and can contribute heavily to plant genome evolution due to their roles in both genome size variation and functional adaption (Vitte and Panaud, 2005). The repetitive DNA accounted for 51.2% (140.2 Mb) of the pomegranate genome assembly (Table 1), higher than that in similarly sized plant genomes such as *Fragaria vesca* (Shulaev *et al*., 2011). Approximately 82.1% of pomegranate repetitive sequences were annotated as transposable elements (TEs), of which the long terminal repeat (LTR) elements were the most abundant (Table S8). Among the five sequenced plant species investigated in this study (Figure S2), the fraction of the genome consisting of LTR retrotransposons increases with the increase of genome sizes from *Arabidopsis thaliana* (~15% LTR retrotransposons) (Arabidopsis Genome Initiative, 2000), to pomegranate (17.4% LTR retrotransposons) and *E. grandis* (~20.7% LTR retrotransposons; Myburg *et al*., 2014). The two major subfamilies of LTRs found in the pomegranate genome are Copia (~5.87% of total TEs) and Gypsy (~11.55%) (Table S8). Kimura distances (K‐values; Kimura 1980) for all Copia and Gypsy LTRs were characterized to estimate the “age” and transposition history of these two LTR lineages. Pomegranate genome only underwent a more recent expansion of Copia and Gypsy. Conversely, both ancient divergent Copia and Gypsy elements with high K‐values as well as recent ones with low K‐values were found in *V. vinifera* (Figure S3). Kimura profiles consistently supported that Copia and Gypsy retrotransposons existed early in the angiosperm history and diverged into heterogeneous subgroups before the modern plant orders arose (Vitte and Panaud, 2005). Moreover, expression of some Copia and Gypsy copies (e.g. Copia‐99 and Gypsy‐14) was significantly (*P* < 0.001) increased during the development of peels or arils (Figure S4), indicating that the divergent fraction of LTR members could be responsible for specific biological processes in plants (Feschotte *et al*., 2002).

**Figure 1:**
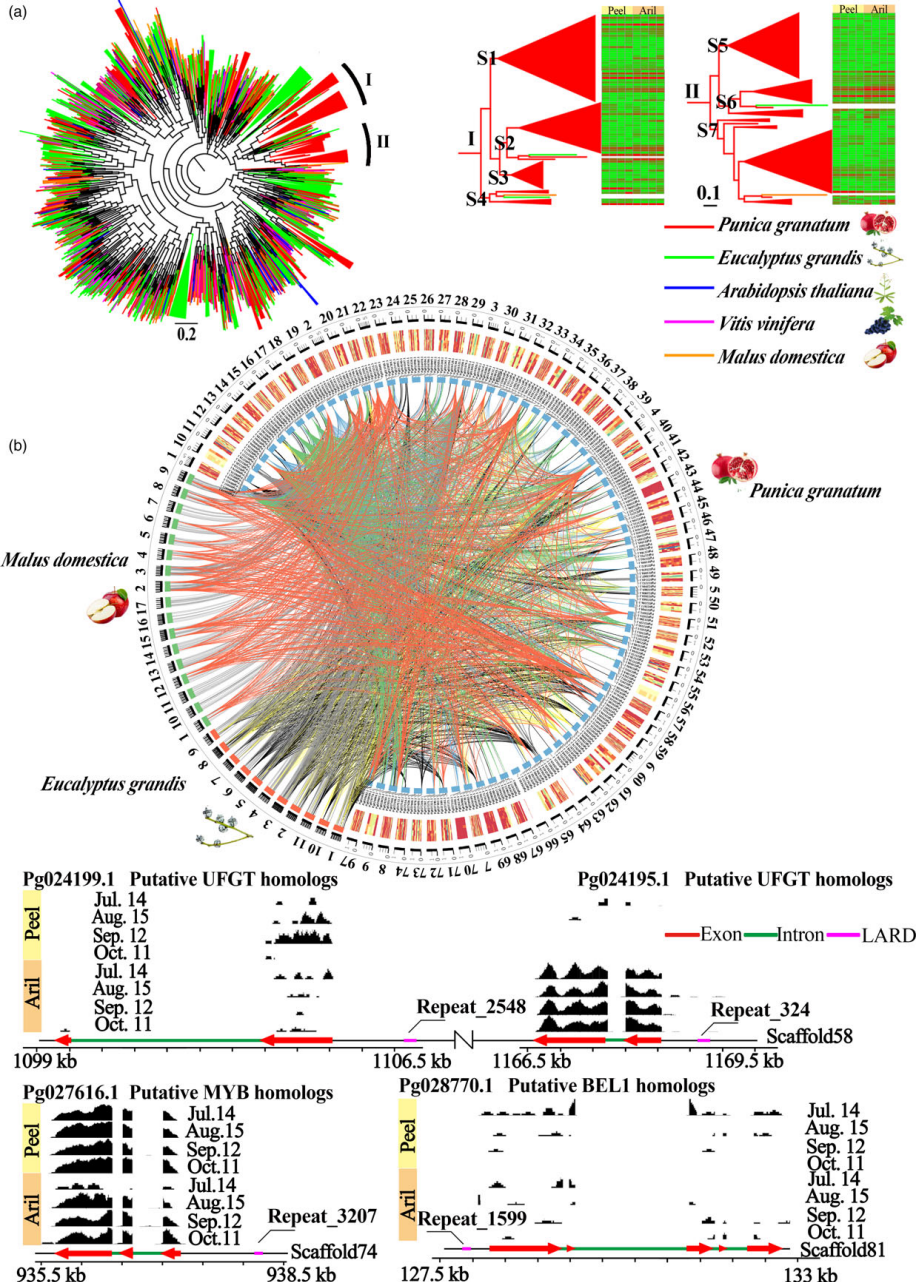
Evolution of large retrotransposon derivatives (LARDs). (a) Phylogenetic tree of LARD families. (b) Genomic circos map of expanded LARDs. Links between species or scaffolds represent the elements from same subclade. Genome regions of four interesting LAPDs and their neighbouring genes are shown at the bottom. Black blocks above the genome regions indicate the cumulative coverage of peel and aril RNA‐Seq data at different developmental stages.

Large retrotransposon derivatives (LARDs) are nonautonomous elements considered to be the remnants of deletion of autonomous LTR retrotransposons. Pan‐plant genomics and Kimura profiles showed that pomegranate possessed the highest ratio of LARDs with low K values (Figures S2 and S3), revealing that pomegranate LARD families might have expanded during recent evolution. A higher ratio of LARDs with high K values in pomegranate than in *Arabidopsis thaliana*, apple and grape (Figure S3) also suggested an ancient retrotransposon activity of pomegranate LARD families. Phylogenetic analysis of LARDs also supported the pomegranate‐lineage‐specific gene radiations (clades I and II; Figure 1a), which could contribute to unique evolutionary changes and novel phenotypic adaptation (Brockington *et al*., 2015). Furthermore, RNA‐Seq analysis of the expanded LARDs (Figure S5) provided a wide and distinct landscape of their expression patterns during the development of peel and aril. For instance, Repeat1156 and Repeat1962 were highly expressed in peel while Repeat684 was highly expressed in aril (Figure S5). Moreover, a comparison of the LARDs in clades I and II in pomegranate, apple and *E. grandis* genomes shows that expanded LARDs in pomegranate were mainly located on scaffolds 2, 4, 18, 22, 23, 33, 51, 57, 58, 64, 69 and 71, with most of them located in the promoter blocks, possibly altering the LARD‐induced alleles of gene expression patterns (Figure 1b). Interestingly, LARDs in scaffold 58 affected the gene expression of putative UDP‐glucose:flavonoid glucosyltransferase (UFGT) homologous genes (*Pg024195.1* and *Pg024199.1*, Figure 1b), which can glycosylate anthocyanidins to anthocyanins (Jaakola, 2013). A LARD element (Repeat3207) in the promoter of a putative MYB paralogue (*Pg027616.1*), which was highly expressed in peel and aril during fruit coloration, was also inferred to be associated with anthocyanin biosynthesis (Figure 1b). Repeat1599 in the promoter of *Pg028770.1* (Figure 1b), a putative BEL1 homologue with a central role in ovule development (Colombo *et al*., 2008), might alter the sequence polymorphism and contribute to the development of a marker for the ovule development. Together, the recent pomegranate‐lineage‐specific radiations of LARDs could be responsible for the specific functional traits in fruit development, such as coloration and ovule development.

### Comparative genomic analysis between pomegranate and other plant species

**Figure 2:**
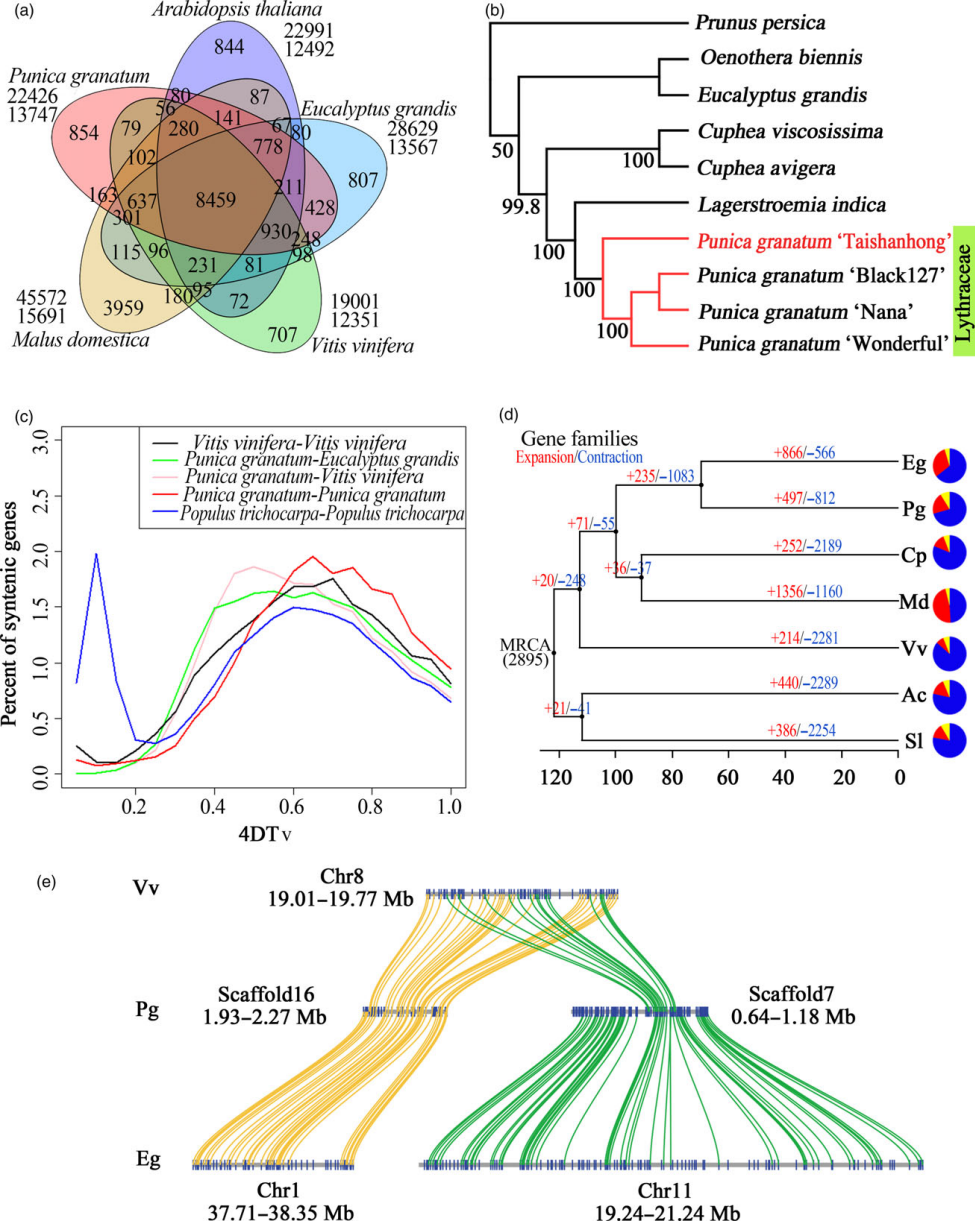
Comparative genomic analysis of pomegranate and other eudicot species. (a) Venn diagram of shared orthologous gene families in pomegranate, *Eucalyptus grandis*,* Malus domestica*,* Vitis vinifera* and *Arabidopsis thaliana*. The gene family number is listed in each component. (b) Phylogenetic tree constructed from 106 single‐copy gene families. (c) Distribution of the 4DTv distance between syntenically orthologous genes. (d) Gene family expansion and contraction analysis. MRCA, most recent common ancestor. Gene family expansions and contractions are indicated by numbers in red and blue, respectively. Blue and red portions of the pie charts represent the contracted and expanded gene families relative to MRCA, respectively, while the grey portions represent the conserved gene families. (e) Schematic diagram of large‐scale duplication events.

A gene family cluster analysis of the complete gene sets of pomegranate, *E. grandis*, apple (*M. domestica*), Arabidopsis (*Arabidopsis thaliana*) and grape (*Vitis vinifera*) was performed. A total of 22 426 genes in the pomegranate genome were grouped into 13 747 gene clusters, of which 8459 were shared by all five species (Figure 2a). Pomegranate shared more gene family clusters with *E. grandis* (11 992) than with any of the other three species, and we also inferred a relatively close taxonomic relationship between these two species from their presence in a shared clade in a phylogenetic tree constructed with 172 single‐copy genes (Figure S6). Furthermore, we assembled the transcriptomes of six species in the Lythraceae family, as well as *Oenothera biennis* (Onagraceae family of the order Myrtales) and then reconstructed a species tree of the Lythraceae family (Figure 2b). On the basis of this tree, four pomegranate cultivars and *Lagerstroemia indica* were classified into one monophyletic clade, and clustered in a group with two species from the *Cuphea* genus. Based on the genomic phylogenetic analysis, we concluded that the *Punica* genus belongs to the Lythraceae family.

We identified 2749 syntenic blocks within the pomegranate genome, and also identified syntenic blocks between the genomes of pomegranate and grape, and pomegranate and *E. grandis*, as well as within the grape and *Populus trichocarpa* genomes. The distribution of 4DTv (transversions at fourfold degenerate sites) of homologous gene pairs within these syntenic blocks suggested that pomegranate has not undergone any recent lineage‐specific whole‐genome duplication (WGD) events, but shared the paleohexaploidy event (γ) of all eudicots (Figure 2c). However, the divergence between pomegranate and *E. grandis*, estimated based on the MCMCtree (Yang, 2007), occurred at ~69.6 (51.5–85.0) million years ago (MYA), after the paleotetraploidy event (109.9 MYA) identified in the *E. grandis* genome (Myburg *et al*., 2014) (Figure 2d), indicating that this WGD event is shared by pomegranate and *E. grandis*. Further analysis of the syntenic blocks between pomegranate and grape, whose genome has not undergone recent genome duplication (Jaillon *et al*., 2007), and pomegranate and *E. grandis* suggested that the majority of grape syntenic regions had two orthologous regions in pomegranate, while the majority of *E. grandis* syntenic regions had one in pomegranate (Figure 2e; Table S9). In addition, *Ks* (synonymous substitution rate) values of syntenic paralogous genes from the ancient duplications within pomegranate and *E. grandis* showed similar distribution patterns (Figure S7). Taken together, these findings strongly support that the paleotetraploidy event identified in *E. grandis* is shared by pomegranate.

We identified 15 gene families that have undergone significant (*P*‐value <0.01) expansion in the pomegranate genome. These families were found to be enriched with genes involved in self‐incompatibility and other specialized biological pathways (Figure S8), suggesting that these pathways have evolved distinctly in pomegranate compared to other plant species.

### Biosynthesis of ellagitannin‐based compounds

**Figure 3:**
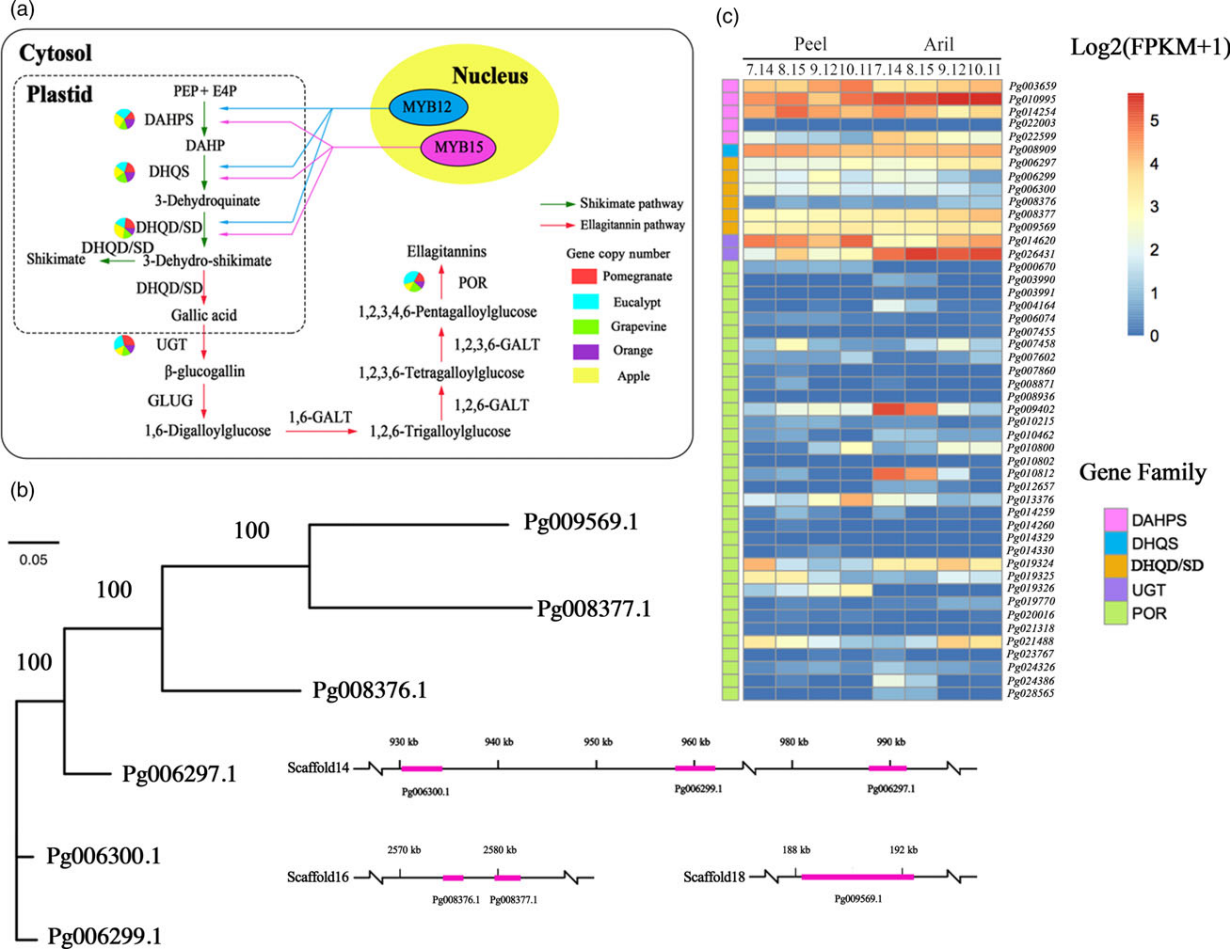
Evolution of ellagitannin biosynthesis in pomegranate. (a) Ellagitannin biosynthetic pathway in pomegranate. Green and red arrows represent the shikimate and ellagitannin pathways, respectively. The numbers of genes in each family in the ellagitannin metabolic pathway in pomegranate, *Eucalyptus grandis*, grape, orange and apple genomes are shown in the pie charts. (b) Phylogenetic analysis and genome locations of DHQD/SD genes in pomegranate. (c) Expression heat map of genes related to the synthesis of ellagitannins in peel and aril during pomegranate fruit development.

To investigate the molecular basis underlying the biosynthesis of the ellagitannin‐based compounds, we performed integrated genomic and transcriptomic analyses of genes in the ellagitannin biosynthetic pathway (Figure 3a; Supporting Information).

The enzyme 3‐dehydroquinate dehydratase/shikimate dehydrogenase (DHQD/SD) serves as a key bridge linking the shikimate pathway and the ellagitannin biosynthetic pathway (Maeda and Dudareva, 2012). Six DHQD/SD genes were identified in the pomegranate genome, of which three (*Pg006297.1*,* Pg006299.1* and *Pg006300.1*) were tandem duplicated and located in a 100‐kb region (Figure 3b). Although all three of these genes were highly expressed in both fruit peels and arils, *Pg006299.1* and *Pg006300.1* showed decreased expression during fruit development (Figure 3c; Figure S9), consistent with the fact that levels of punicalagin, ellagic acid and gallic acid also decreased during pomegranate fruit development (Han *et al*., 2015), indicating their potential roles in ellagitannin biosynthesis. Two other DHQD/SD genes, *Pg008377.1* and *Pg008376.1*, were also tandem duplicated. *Pg008377.1* was highly expressed in fruits while *Pg008376.1* exhibited a very low expression level (Figure 3c), suggesting their subfunctionalization after the tandem duplication. In addition, two UDP‐glucose:gallate glucosyltransferase (UGT) genes (*Pg014620.1* and *Pg026431.1*) were identified in the pomegranate genome and they showed distinct expression patterns: *Pg014620.1* was expressed higher in peel than in aril, while *Pg026431.1* was expressed higher in aril than in peel (Figure 3c; Figure S9), suggesting the tissue‐specific roles of these two genes in the ellagitannin biosynthesis.

Another key enzyme family in the ellagitannin biosynthetic pathway is pentagalloylglucose oxygen oxidoreductase (POR). A total of 34 POR genes were identified in the pomegranate genome (Table S10). Phylogenetic analysis placed these genes into twelve groups, and member expansion was observed in group 1 (Figure S10). Four genes in group 1 (*Pg007458.1*,* Pg019324.1*,* Pg019325.1* and *Pg021488.1*) were highly expressed in both fruits and arils, and the expression of *Pg019324.1* and *Pg019325.1* showed a decreased pattern in the peel during fruit development (Figure S9). Of genes in other groups, *Pg009402.1* and *Pg010812.1* showed a clear descending expression pattern in aril during fruit development (Figure S9). Reduced expression of these genes during peel and aril development could be responsible for the decreased productions of punicalagin, ellagic acid and gallic acid (Han *et al*., 2015).

Interestingly, sequence homology searches did not reveal any genes predicted to encode β‐glucogallin O‐galloyltransferase (GLUG) or galloyltransferase (GALT), known enzymes in the ellagitannin biosynthetic pathway. These genes may have diverged to such a degree in pomegranate that sequence homology has been lost or pomegranate may have developed alternative reactions for the steps catalysed by these two enzymes.

### Evolution of the anthocyanin biosynthetic pathway

**Figure 4:**
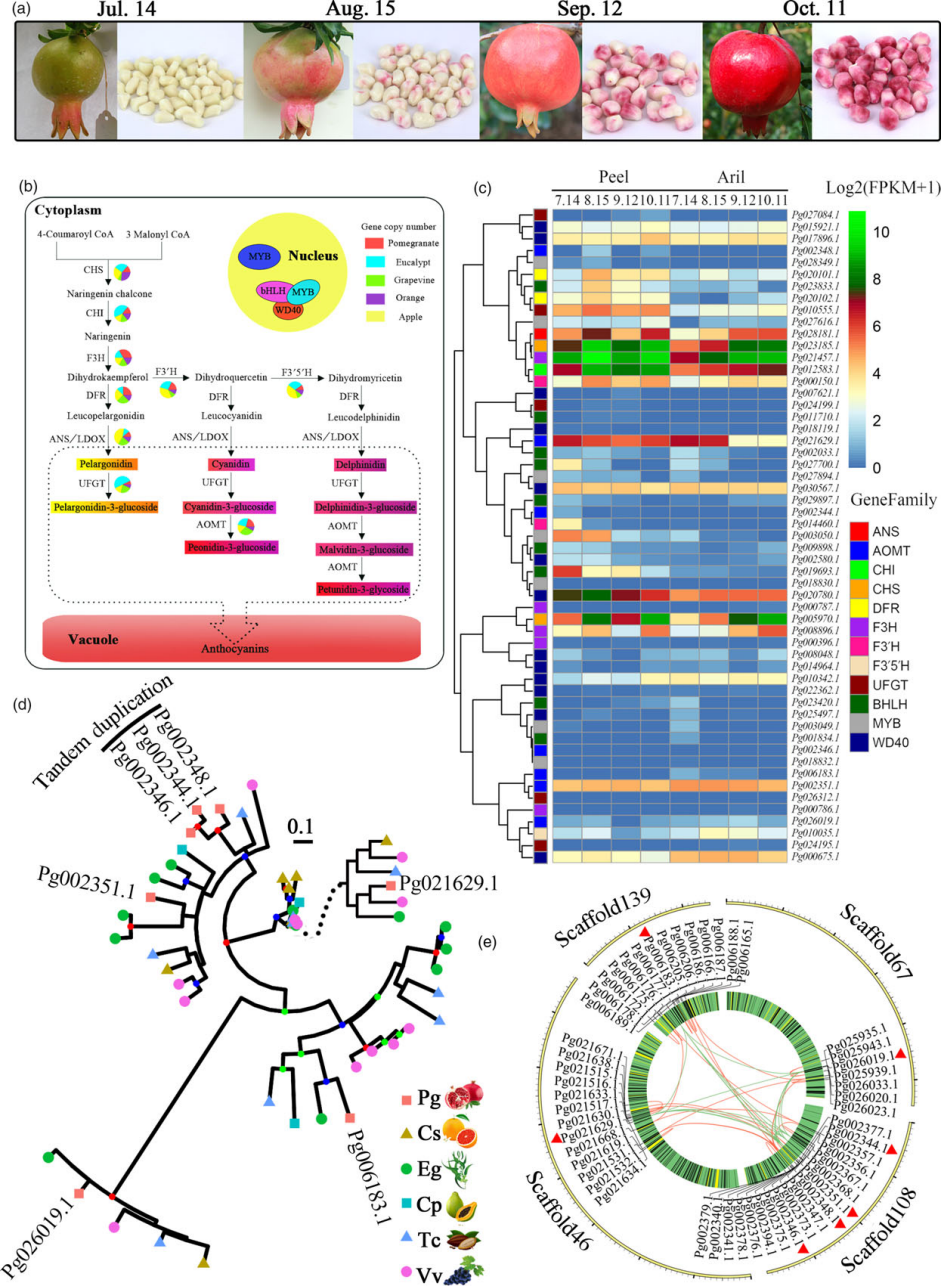
Anthocyanin biosynthetic pathway in pomegranate. (a) Fruits and arils of ‘Taishanhong’ pomegranate at different developmental stages. (b) Anthocyanin biosynthetic pathway in pomegranate. The numbers of genes in each family in the anthocyanin biosynthetic pathway in pomegranate, *Eucalyptus grandis*, grape, orange and apple are shown in the pie charts. (c) Expression heat map of genes related to the synthesis of anthocyanins in peel and aril during fruit colour development. (d) Phylogenetic analysis of the AOMT genes in plants within the Malvids clade and the outgroup species, grape. (e) Genome location of AOMT genes.

Anthocyanins are the major pigments responsible for the colour of pomegranate fruits (Ben‐Simhon *et al*., 2015). Unlike other fruits such as *Litchi chinensis* (Hu *et al*., 2016) and *V. vinifera* (Boss *et al*., 1996), both peel and aril in pomegranate are bright red at the ripe stage (Figure 4a). Although the anthocyanin biosynthetic pathway in fruit peels has been studied in several species (Jaakola, 2013), it has not been well characterized in arils. From our genome assembly, 26 anthocyanin biosynthesis genes from 12 families were identified (Figure 4b; Table S11). The wide diversity of anthocyanin compounds comes from the glycosylation (Montefiori *et al*., 2011) and methylation (Roldan *et al*., 2014) of the basic flavonol structure. In the anthocyanidin biosynthetic pathway, members of each enzyme had substantial expression in both peel and aril, and most of them had preferential expression in peel (Figure 4c; Figure S11). By contrast, for anthocyanidin modification only three genes (*Pg010555.1*,* Pg002351.1* and *Pg021629.1*) were highly expressed in peel and aril (Figure 4c). High‐performance liquid chromatography (HPLC) analyses showed that the total anthocyanin content in peel (~118.65 mg/100 g) was higher than that in aril (~36.41 mg/100 g) (Zhu *et al*., 2015). Our results support the tissue‐specific expression pattern for anthocyanin biosynthesis and indicate that highly up‐regulated expression during fruit development of genes encoding enzymes such as chalcone synthase (CHS), chalcone isomerase (CHI), flavonoid 3‐hydroxylase (F3H), flavonoid 3′‐hydroxylase (F3′H), dihydroflavonol 4‐reductase (DFR), anthocyanidin synthase/leucoanthocyanidin dioxygenase (ANS/LDOX), UDP‐glucose:flavonoid glucosyltransferases (UFGT) and anthocyanin O‐methyltransferase (AOMT) could be responsible for the skin and aril colour transition from white to red (Figure 4a) (Zhao *et al*., 2015).

The pomegranate and grape genomes have an identical number of copies (7) of anthocyanin AOMT genes, but higher than other three species (Table S11), for example, six in *E. grandis* (Myburg *et al*., 2014) and only two in *C. sinensis* (Xu *et al*., 2013). AOMTs catalyse the final step of the anthocyanin biosynthesis pathway by mediating the methylation of anthocyanins (Roldan *et al*., 2014). High copy number of AOMT genes in fruit species with diverse anthocyanins supports a putative link between the expansion of the AOMT family and the ability to produce anthocyanins. The divergent AOMTs are inferred to be responsible for distinct colours of pomegranate fruits. Phylogenetic analysis of pomegranate AOMTs and their homologs from six other plant species within the Malvidae clade revealed one recent AOMT gene expansion in the pomegranate genome, comprised of three tandem duplicated genes (Figure 4d). The genes flanking the 200‐kb upstream and downstream regions of the three AOMTs were also expanded (Figure 4e), revealing that the AOMT tandem duplication results from the large‐scale duplication in the pomegranate genome. The seven AOMTs in pomegranate exhibited distinct expression patterns during fruit development, among which *Pg002351.1* was highly expressed in peel and aril during fruit development (Figure 4c; Figure S11). *Pg021629.1* was down‐regulated during fruit early development in peel and aril. The tissue‐specific expression patterns of AOMTs could responsible for anthocyanin accumulation in peel and aril. Together, the tandem duplicated AOMT genes might have evolved independently in anthocyanin biosynthesis.

Anthocyanin biosynthetic genes are activated by a transcriptional activation complex (the MBW complex) consisting of R2R3‐MYB, BHLH and WD40 proteins (Jaakola, 2013). In *Arabidopsis*, genes encoding enzymes in the early steps of the anthocyanin biosynthetic pathway that lead to the production of flavonols are activated by three R2R3‐MYB regulatory genes (*AtMYB11*,* AtMYB12* and *AtMYB111*), whereas the activation of the late biosynthetic genes, leading to the production of anthocyanins, requires an MBW complex (Petroni and Tonelli, 2011). In the pomegranate genome, we identified seven R2R3‐MYB genes, nine BHLH genes and 13 WD40 genes that were highly expressed in both peel and aril (Figure 4c), suggesting their roles in regulating anthocyanin production in pomegranate fruit. Recently, an NAC transcriptional factor BLOOD (BL) was found to up‐regulate the accumulation of anthocyanin in peach (Zhou *et al*., 2015). However, BLAST searches using this gene yielded no significant hits in the pomegranate genome assembly, indicating possible different mechanisms of anthocyanin biosynthesis between these two species.

### Ovule developmental biology

The polycaryoptic trait is a common target of the plant breeding programmes. In pomegranate, more than one hundred ovules can grow in a single ovary, and they develop into seeds with arils, which consist of epidermal cells derived from the integument (Dahlgren and Thorne, 1984). Compared to cucumber and tomato, which have parietal (Schaefer and Renner, 2011) and axial (Zhang *et al*., 1994) placentas, respectively, pomegranate carpels become superposed into two or three layers by differential growth, the lower comprised of axial placentas and the upper ostensibly parietal placentas (Teixeira da Silva *et al*., 2013). Consequently, pomegranate represents a unique system for studying ovule developmental biology.

We identified and compared genes involved in the ovule development from the genomes of pomegranate, castor bean [another species with arils (Chan *et al*., 2010)], cucumber and tomato. The pomegranate genome has 237 candidate genes belonging to twelve families associated with ovule development (Figure S12). The AG clade, including the AG, SEP, SHP and STK families, had the largest copy number (39) in the pomegranate genome (Figure S12). AG‐clade genes are required for specifying the ovule identity (Brambilla *et al*., 2007; Colombo *et al*., 2008), suggesting that the expansion of AG‐clade genes might play an important role in the development of the pomegranate‐specific type of ovules. Furthermore, structure and transcriptome analyses showed that the BEL1 gene (*Pg029909.1*) could be functionally inactive due to a frameshift mutation, and this gene exhibited a low expression level. BEL1 genes had a negative role in regulating the WUS expression, resulting in carpelloid structures (Colombo *et al*., 2008). Low copy and pseudogenization of the BEL1 genes suggest a possible links between the contraction and inactivation of the BEL1 family and the multicarpel formation. Additionally, the pomegranate genome also has a higher copy number (87) of CUC genes than the other three genomes (Figure S12). CUC proteins have been reported to regulate ovule production (Duszynska *et al*., 2013), and expansion of the CUC family in the pomegranate genome may be a key factor in the production of the large number of ovules (Duszynska *et al*., 2013). Based on our comparative genomic analysis, the pomegranate‐specific ovule development and the polycaryoptic phenotype can likely be attributed to the expansions of the AG and CUC families and the contraction and inactivation of the BEL1 family.

## Discussion

A high‐quality genome sequence of pomegranate was assembled, which offers a valuable resource for resolving the previously debated taxonomic status of the *Punica* genus (Berger *et al*., 2016). *Punica* was previously considered a member of the monogeneric Punicaceae family (Narzary *et al*., 2010) but was later moved into the Lythraceae family (Berger *et al*., 2016; Byng *et al*., 2016). Our phylogenomic analysis strongly supports this reclassification of *Punica* into Lythraceae. Consequently, pomegranate represents the first species in the Lythraceae family that has a sequenced genome, providing an important reference for future comparative and evolutionary genomics studies.

The pomegranate fruit is highly enriched in ellagitannin‐based compounds, which are known to possess antioxidant activities (Johanningsmeier and Harris, 2011). Another important fruit quality trait of pomegranate is the colour formation related to the anthocyanin biosynthesis in the peel and aril. Our genomic and RNA‐Seq analyses offer deeper insights into the molecular basis underlying the ellagitannin and anthocyanin biosynthesis in pomegranate. Several key gene families in each of the associated pathways were found to have undergone tandem duplications and specific family members showed differential expression patterns in the peel and/or aril during fruit development, indicative of their important roles in the production of these compounds.

With hundreds of ovules in a single ovary (Teixeira da Silva *et al*., 2013), rare heterotypic placentation and arils developed from integuments (Dahlgren and Thorne, 1984), pomegranate has provided a unique system for studying the ovule development. Our comparative genomic analysis provided evidence that the pomegranate‐specific ovule development and the polycaryoptic phenotype can be attributed, at least in part, to the expansions of the MADS‐box AG clade and the CUC family, respectively.

In summary, the pomegranate genome represents an invaluable resource for the genetic improvement of the crop and for better understanding of the genome evolution. Genetic markers can be developed based on this genome sequence, for further studies involving genetic map construction, positional cloning, strain identification and marker‐assisted selection, which will collectively accelerate pomegranate breeding.

## Materials and methods

### Sample preparation and sequencing

Genomic DNA was extracted from the leaves of *P. granatum* ‘Taishanhong’, using the CTAB protocol. Paired‐end and mate‐pair Illumina genome libraries with insert sizes ranging from 220 bp to 17 kb were constructed using the NEB Next Ultra DNA Library Prep Kit (NEB, Ipswich, MA, USA) and sequenced on a HiSeq 2500 system (Illumina, San Diego, CA, USA) according to the manufacturer's instructions. Raw reads were processed to remove low‐quality and adaptor sequences, and to collapse duplicated reads using NxTrim (O'Connell *et al*., 2015).

### *De novo* genome assembly

The high‐quality cleaned reads were assembled *de novo* using ALLPATHS‐LG (Butler *et al*., 2008), and the mate‐pair reads were then used to construct scaffolds, using SSPACE2.0 (Boetzer *et al*., 2011). Gap filling was performed using GapCloser provided in SOAPdenovo2 (Luo *et al*., 2012). Assembled scaffolds were compared against the Genbank nt database using megablast and against a set of known microbial proteins using BLASTX. Scaffolds classified as microbial sequences, unanchored rDNA, mitochondrion, chloroplast and repetitive sequences, as well as those <1 kb were removed from the final assembly.

### Repeat annotation

We first identified repeat sequences in the *P. granatum* genome using the *de novo* prediction programs, LTR_FINDER (Xu and Wang, 2007), MITE‐Hunter (Han and Wessler, 2010), RepeatScout (Price *et al*., 2005) and PILER‐DF (Edgar and Myers, 2005) and then classified the identified repeat sequences with PASTEClassifier (v1.0; Wicker *et al*., 2007). The classified repeat sequences and the Repbase database (Bao *et al*., 2015) were combined to construct a nonredundant repeat sequence library. RepeatMasker (v4.0.6; http://www.repeatmasker.org) was used to identify the *P. granatum* repeat sequences based on the constructed repeat sequence library. We also analysed the divergence rate of the TE elements in pomegranate, Arabidopsis, apple, grape genomes using both the Repbase and the RepeatModeler TE libraries. The divergence rate was calculated between the identified TE elements in the genome and the consensus sequence in the TE library (Repbase or RepeatModeler).

### Gene prediction and annotation

The repeat‐masked *P. granatum* genome sequence was used for gene prediction with the following methods: (i) *ab initio* gene prediction, (ii) homologous sequence searching, (iii) transcriptome sequence mapping. We first assembled the RNA‐Seq reads into contigs using Trinity (v2.1.1; Haas *et al*., 2013). The *P. granatum*‐specific parameter file was trained by the *ab initio* gene prediction software Augustus (v1.0.2; Stanke *et al*., 2006) using the *bona fide* gene models, which were identified from the assembled RNA‐Seq contigs by PASA (v1.2; Campbell *et al*., 2006). Using this parameter file, we performed *ab initio* gene predictions using Augustus, SNAP (Korf, 2004) and GlimmerHMM (v0.5.9; Majoros *et al*., 2004), respectively. We also performed *ab initio* gene predictions using Genscan (v0.5.9; Burge and Karlin, 1997) and GeneID (v1.4; Parra *et al*., 2000) with the Arabidopsis parameter file. In homologous sequence searches, we aligned the protein sequences from *E. grandis*, the plant‐specific UniProtKB/Swiss‐Prot database (Schneider *et al*., 2004) and the GenBank nr database against the *P. granatum* genome using TBLASTN with a sequence identity >50% and an E‐value cut‐off of 1E‐5. GeneWise (Birney *et al*., 2004) was then used to extract the accurate exon‐intron information. GMAP (v1.0.0; Wu and Watanabe, 2005) was used to align the assembled RNA‐Seq contigs to the *P. granatum* genome. Finally, we generated an integrated gene set using GLEAN (Elsik *et al*., 2007).

Functional annotation of the predicted genes was performed by comparing their protein sequences against a number of protein sequence databases, including GenBank nr, COG (Tatusov *et al*., 2000), KEGG (Kanehisa and Goto, 2000) and TrEMBL (Bairoch and Apweiler, 1997), using BLASTP with an E‐value cut‐off of 1E‐5.

### Collinearity and WGD

All‐against‐all BLASTP analyses of protein sequences were performed between *P. granatum*,* V. vinifera*,* E. grandis* and *P. trichocarpa* using an E‐value cut‐off of 1E‐10. Syntenic regions within and between species were identified using MCScan (Wang *et al*., 2012) based on the BLASTP results. A syntenic region was identified if it contained a minimum of 10 and a maximum of 25 genes in the identified gene pairs. Protein sequences of homologous gene pairs in the identified syntenic regions were aligned by MUSCLE (v3.8.31; Edgar, 2004), and the protein alignments were then converted to coding sequence (CDS) alignments. The 4DTv value of each gene pair was calculated using the sum of transversions of fourfold degenerate sites divided by the sum of fourfold degenerate sites and then corrected using the HKY model (Hasegawa *et al*., 1985). The Ks value of each syntenic gene pair was calculated using the Yn00 program in the PAML package (Yang, 2007).

### Gene family evolution and phylogenetic analyses

Protein sequences of *P. granatum*,* E. grandis*,* M. domestica*,* A. thaliana* and *V. vinifera* were used in an all‐against‐all BLASTP analysis. The results were analysed using the OrthoMCL software (Li *et al*., 2003) with an MCL inflation parameter of 1.5 to identify gene family clusters. Gene family clusters were also identified among *P. granatum*,* E. grandis*,* M. domestica*,* C. papaya*,* V. vinifera*,* S. lycopersicum* and *A. chinensis*. Single‐copy gene clusters shared by all seven species were identified and used to construct a phylogenetic species tree using PhyML (v3.0; Guindon *et al*., 2010). The divergence time was estimated by MCMCtree (Yang, 2007) using the known divergence time of *V. vinifera* and *M. domestica*, and *V. vinifera* and *A. chinensis* from the TimeTree database (Hedges *et al*., 2006). In addition, we used a Pfam domain‐based method to infer the gene family expansions as described in Albertin *et al*. (2015).

To determine the taxonomic position of *P. granatum*, transcript assemblies were performed using Trinity (Haas *et al*., 2013) using RNA‐Seq reads from other *P. granatum* cultivars and other species (three cultivars of *P. granatum*: ‘Black127’, ‘Nana’, and ‘Wonderful’; three species from the Lythraceae family: *Lagerstroemia indica*,* Cuphea viscosissima* and *Cuphea avigera*; and one species from the Onagraceae family: *Oenothera biennis*) downloaded from the NCBI sequence archive (SRA) database with the following accession numbers: pomegranate ‘Black127’, SRX395468; pomegranate ‘Nana’, SRX395465; pomegranate ‘Wonderful’, SRX034876; *Lagerstroemia indica*, SRX470007; *Cuphea viscosissima*, SRX1361461; *Cuphea avigera*, SRX1361546; *Oenothera biennis*, ERX651036, ERX651029, ERX651035, ERX651028 and ERX651064. The open‐reading frame (ORF) of each assembled unigene was identified using GeneMarkS‐T (Besemer *et al*., 2001) and the translated amino acid sequences were then used for phylogeny reconstruction. An all‐against‐all BLASTP analysis was performed with the cut‐off E‐value <10^−4^, and orthologous gene families were then constructed using OrthoMCL (Li *et al*., 2003). A total of 106 single‐copy gene families were obtained. Multiple alignment of protein sequences in each gene family was performed using Muscle (Edgar, 2004). A maximum‐likelihood (ML) tree was constructed using PhyML (v3.1; Guindon *et al*., 2010) with the JTT model and bootstrap repeat of 1000.

### RNA collection and sequencing

Pomegranate has a long florescence time, with fruit setting being stable about two months after pollination. To investigate the transcriptomic landscape related to the traits of fruit quality development, peel and aril samples were collected from four different developmental stages of pomegranate fruits, two months after pollination (July 14; fruits began to colour and accumulated components with high antioxidant activity), three months after pollination (Aug. 15; fruit colour changed obviously and the content of antioxidant components decreased), four months after pollination (September 12; fruit colour change significantly) and five months after pollination (October 11; Ripe), in 2015. Three biological replicates were analysed for each sample. Total RNA was extracted using TRI reagent (Sigma‐Aldrich, St. Louis, MO, USA) according to the manufacturer's instructions. RNA‐Seq libraries were constructed using the NEB Next UltraTM RNA Library Prep Kit (NEB) and sequenced on an Illumina HiSeq 4000 platform (Illumina) according to the manufacture's protocols.

### Quantification and differential gene expression analysis

Paired‐end RNA‐Seq reads were processed to remove adaptor sequences and low‐quality reads and then mapped to the *de novo*‐assembled pomegranate genome sequence using TopHat2 (v2.0.13; Trapnell *et al*., 2012) with default parameters. Cufflinks (v2.2.1; Trapnell *et al*., 2012) was used to assemble the mapped reads for each sample. The assembled contigs were then merged with the reference gene annotation into a unified annotation, which was used to quantify gene expression in each sample. We used the FPKM (fragments per kilobase exon model per million mapped fragments) as the normalized gene expression level. Differentially expressed genes across different developmental stages were identified using DESeq (v1.20.0; Anders and Huber, 2010). Genes with an adjusted *P*‐values <0.01 were considered to be differentially expressed. The expression of transposable elements was derived from the RNA‐Seq data using kallisto (v0.431; Bray *et al*., 2016).

### Accession codes

The pomegranate whole‐genome sequence has been deposited in GenBank under a BioProject with accession number PRJNA355913.

## Author contributions

T.Z., C.L., W.X., H.X., J.Z., Y.L., Y.C., M.Y., X.H. and H.W. contributed to plant sample collection; T.Z., F.H., M.L., W.Z., D.G., Y.X. and D.L. worked on genomic DNA sequencing and genome assembly; T.Z., Z.F., F.H., C.L., W.X., W.Z., M.Z., Y.J., H.X., H.D. and L.W. contributed to transcriptome sequencing and gene expression analyses; T.Z., Z.F., F.H., S.W., W.Z. and Y.J. conducted genome annotation and comparative genomic analyses; T.Z., Z.F. and F.H. wrote and revised the manuscript; Z.Y., Y.F. and H.Z. conceived and manage the project and designed experiments. All authors read and approved the manuscript.

## Supporting information


**Figure S1** 17‐mer frequency distribution of sequence reads from the library with insert sizes of ~220 bp.
**Figure S2** Comparison of repeat sequences in pomegranate and other plant species.
**Figure S3** Distribution of divergence rates of three types of TEs in the genomes of *Punica granatum* (a), *Eucalyptus grandis* (b), *Malus domestica* (c), *Vitis vinifera* (d) and *Arabidopsis thaliana* (e).
**Figure S4** Expression profiles (TPM; transcripts per million) of a subset of Copia (a) and Gypsy (b) retrotransposons in the peel and aril during pomegranate fruit development.
**Figure S5** Expression profiles of large retrotransposon derivatives (LARDs) in the peel and aril during pomegranate fruit development.
**Figure S6** Maximum likelihood (ML) phylogenetic tree of pomegranate and other plant species constructed using single‐copy genes.
**Figure S7** Distribution of synonymous substitutions rates (*Ks*) of syntenic gene pairs within *Punica granatum* and *Eucalyptus grandis*.
**Figure S8** Expanded gene families in the pomegranate genome.
**Figure S9** Expression profiles of the ellagitannin biosynthetic genes in the peel and aril during pomegranate fruit development.
**Figure S10** Phylogenetic tree of pentagalloylglucose oxygen oxidoreductase (POR) genes in pomegranate (*Punica granatum*), grape (*Vitis vinifera*), orange (*Citrus sinensis*), papaya (*Carica papaya*) and tomato (*Solanum lycopersicum*).
**Figure S11** Expression profiles of the anthocyanin biosynthetic genes in the peel and aril during pomegranate fruit development.
**Figure S12** Regulation of ovule development in pomegranate.
**Table S1** Statistics of the genome sequencing data.
**Table S2** Pomegranate genome size estimated by flow cytometry.
**Table S3** Statistics of the final genome assembly.
**Table S4** Coverage of expressed sequence tags (ESTs) by the assembled pomegranate genome.
**Table S5** Coverage of unigenes assembled from the RNA‐Seq data by the assembled pomegranate genome.
**Table S6** Functional annotation of the predicted protein‐coding genes in pomegranate.
**Table S7** Non‐coding RNAs predicted in the pomegranate genome.
**Table S8** Classification of pomegranate repeat sequences.
**Table S9** Syntenic comparisons between pomegranate, grape and *Eucalyptus grandis* genomes.
**Table S10** Number of ellagitannin biosynthetic genes identified in each family in pomegranate and other plant species.
**Table S11** Number of anthocyanin biosynthetic genes identified in each family in pomegranate and other plant species.
