# Supplementary material for: The pomegranate (Punica granatum L.) genome provides insights into fruit quality and ovule developmental biology
Source: Plant Biotechnol J. 2018 Jan 22;16(7):1363–74. doi: 10.1111/pbi.12875 (PMC5999313; doi:10.1111/pbi.12875)
Supplement: Supplementary file 1 — Figure S1 17‐mer frequency distribution of sequence reads from the library with insert sizes of ~220 bp. Figure S2 Comparison of repeat sequences in pomegranate and other plant species. Figure S3 Distribution of divergence rates of three types of TEs in the genomes of Punica granatum (a), Eucalyptus grandis (b), Malus domestica (c), Vitis vinifera (d) and Arabidopsis thaliana (e). Figure S4 Expression profiles (TPM; transcripts per million) of a subset of Copia (a) and Gypsy (b) retrotransposons in the peel and aril during pomegranate fruit development. Figure S5 Expression profiles of large retrotransposon derivatives (LARDs) in the peel and aril during pomegranate fruit development. Figure S6 Maximum likelihood (ML) phylogenetic tree of pomegranate and other plant species constructed using single‐copy genes. Figure S7 Distribution of synonymous substitutions rates (Ks) of syntenic gene pairs within Punica granatum and Eucalyptus grandis. Figure S8 Expanded gene families in the pomegranate genome. Figure S9 Expression profiles of the ellagitannin biosynthetic genes in the peel and aril during pomegranate fruit development. Figure S10 Phylogenetic tree of pentagalloylglucose oxygen oxidoreductase (POR) genes in pomegranate (Punica granatum), grape (Vitis vinifera), orange (Citrus sinensis), papaya (Carica papaya) and tomato (Solanum lycopersicum). Figure S11 Expression profiles of the anthocyanin biosynthetic genes in the peel and aril during pomegranate fruit development. Figure S12 Regulation of ovule development in pomegranate. Table S1 Statistics of the genome sequencing data. Table S2 Pomegranate genome size estimated by flow cytometry. Table S3 Statistics of the final genome assembly. Table S4 Coverage of expressed sequence tags (ESTs) by the assembled pomegranate genome. Table S5 Coverage of unigenes assembled from the RNA‐Seq data by the assembled pomegranate genome. Table S6 Functional annotation of the predicted protein‐coding genes in pomegranate. Table S [file PBI-16-1363-s001.docx]

**Supplemental Note**

**Significance and impact of pomegranate**

Pomegranate (*Punica granatum* L.) is an ancient fruit crop that dates back to the middle Eocene [48.6-40.4 million years ago (Mya)] as indicated by fossil record (Graham, 2013). However, the taxonomy of pomegranate remains poorly understood, in spite of numerous phylogenetic studies. It has been suggested that pomegranate belongs to the monogeneric family Punicaceae (Narzary *et al.*, 2010), while morphological analysis of ovary, fruit and seed for the Lythraceae family suggests that the family includes the Punicaceae family (Graham and Graham, 2014). The Lythraceae is a large family in the order Myrtales, containing 31 genera and 625-650 species that are widespread in tropical regions, but less common in temperate regions (Qin *et al.*, 2007). Recent molecular phylogenetic studies have also indicated that the Lythraceae family contains the Punicaceae family. A phylogenetic tree of 102 taxa across the Myrtales was reconstructed using sequences from six loci (*rbcL*, *ndhF*, *matK*, *matR*, 18S, and 26S), which classified *Punica* into the Lythraceae clade (Berger *et al.*, 2016). In the Angiosperm Phylogeny Group (APG) IV system, *Punica* is classified as a genus of the Lythraceae family (Byng *et al.*, 2016). In this study we reconstructed a genomic phylogenetic tree, in which pomegranate was also clustered into the Lythraceae clade.

Pomegranate is emerging as a fruit of economic importance worldwide. It is native to central Asia, with China, India, Iran, Turkey and USA being the leading producers (Holland *et al.*, 2009). The annual world production is approximately 3 million tons, with an estimated revenue of over $35,000/ha. Commercial use of pomegranate fruit, including juices, tubs of grains, and dehydrated seeds has contributed to the increase of its cultivated area (Melgarejo-Sanchez *et al.*, 2015). However, this continuing increase in planted acreage is driving a demand for new cultivars. The pomegranate genome sequence presented here provides a valuable resource for facilitating molecular breeding, which will in turn benefit the pomegranate industry worldwide.

Pomegranate is well known as a medicinal plant whose fruits are enriched with compounds that have strong antioxidant activities (Halvorsen *et al.*, 2002; Teixeira da Silva *et al.*, 2013; Trottier *et al.*, 2010). Ellagitannin-based compounds, such as punicalagins, punicalins, gallagic acid, and ellagic acid, can reduce incidences of cardiovascular disease, diabetes, and prostate cancer (Johanningsmeier and Harris, 2011), and represent a major proportion of the pool of antioxidant compounds in the pomegranate fruit (Halvorsen *et al.*, 2002). The concentrations of punicalagin and other ellagitannin-based compounds in the fruit peel are higher than in the aril, and decrease as the fruit ripens (Han *et al.*, 2015). Despite numerous reports regarding extracts and functional verification of punicalagins, punicalins, and other components, few studies have focused on their molecular metabolic pathways. The ellagitannin biosynthetic pathway shares the early steps of the shikimate pathway, which leads to the biosynthesis of phenylpropanoids (Maeda and Dudareva, 2012). The enzyme 3-dehydroquinate dehydratase/shikimate dehydrogenase (DHQD/SD) is bifunctional in that it converts 3-dehydroquinate to 3-dehydro-shikimate, and further catalyzes 3-dehydroshikimate to produce shikimate, as well as synthesizing gallic acid, which serves as a precursor for ellagitannin-based compounds (Maeda and Dudareva, 2012). Gallic acid is then converted to β-glucogallin, catalyzed by UDP-glucose:gallate glucosyltransferase (UGT). Overexpression and suppression by RNAi of UGT84A23 or UGT84A24 in pomegranate hairy root lines did not lead to obvious changes in punicalagin levels; however suppressing the expression of both UGT genes resulted in substantially reduced levels of punicalagin (Ono *et al.*, 2016). POR (pentagalloylglucose oxygen oxidoreductase) regulates the final step of the ellagitannin biosynthesis pathway, leading to the production of diverse ellagitannin-based compounds. Oxidation of 1,2,3,4-penta-O-galloyl-ß-D-glucopiranose to synthesize ellagitannin is catalyzed by POR proteins, which have similar activities to laccase (EC:1.10.3.2) type phenol oxidases (Ascacio-Valdes *et al.*, 2011). However, key steps contributing to the accumulation of ellagitannins have yet to be identified. Here, our integrated genomic and transcriptomic analyses provided a deeper understanding of the regulation of the ellagitannin biosynthetic pathway in pomegranate and the production of punicalagin.

Peel and aril color, due to the accumulation of anthocyanins, is a critical trait in determining pomegranate fruit value and quality. Previous studies have shown that the anthocyanin biosynthetic pathway of pomegranate is highly conserved with that of other fruit trees (Ono *et al.*, 2011), and a detailed pathway was reconstructed based on RNA-Seq (Ono *et al.*, 2011) and qRT-PCR (Zhao *et al.*, 2015) analyses. Anthocyanin composition is mainly affected by the expression of genes encoding flavonoid 3’-hydroxylase (F3’H), flavonoid 3’5’-hydroxylase (F3’5’H), and anthocyanin O-methyltransferase (AOMT) (Azuma *et al.*, 2015). Chalcone synthases (CHS), chalcone isomerase (CHI), flavonoid 3-hydroxylase (F3H) and F3’H constitute the early biosynthetic genes (EBGs) of the anthocyanin biosynthesis pathway, while F3’5’H, dihydroflavonol 4-reductase (DFR), anthocyanidin synthase/leucoanthocyanidin dioxygenase (ANS/LDOX), and UDP-glucose:flavonoid glucosyltransferases (UFGT) make up the late biosynthetic genes (LBGs) (Xu *et al.*, 2015). EBGs are activated by independent and functionally redundant R2R3-MYB regulatory genes, whereas the regulation of LBGs requires a ternary complex of the MYB-bHLH-WD40 transcription factors (MBW complex)(Petroni and Tonelli, 2011). However, very few reports (Hu *et al.*, 2016) have described the pathway in aril, the edible part of the fruit. The integrated genomic and transcriptomic analysis presented here provides a more comprehensive understanding of anthocyanin biosynthesis in both the peel and the aril.

Pomegranate also provides an ideal system for studying ovule developmental biology as it is polycaryoptic, a trait that is valuable in crop production. More than one hundred ovules grow in one pomegranate ovary, and carpels become superposed into two or three layers by differential growth, the lower with axial placentas, the upper with ostensibly parietal placental (Teixeira da Silva *et al.*, 2013). The MADS-box, Homeobox, and AP2-like gene families play key roles in plant ovule development (Kelley and Gasser, 2009; Pinyopich *et al.*, 2003), where AG-MADS transcription factors determine ovule identities and WUS homeobox proteins play crucial roles in ovule cell differentiation. BEL1 proteins restrain the gene expression of WUS to balance the carpel and ovule development. Despite of a detailed knowledge base of ovule developmental biology based on model species like Arabidopsis (Colombo *et al.*, 2008), there have been few equivalent studies of pomegranate. Our comparative genomic study provides a foundation for studying pomegranate seediness biology.

In summary, pomegranate is an ancient medicinal fruit crop with growing economic value, and the first species in the Lythraceae family with a sequenced genome. It provides a unique system for studying the metabolism of ellagitannin-based compounds, fruit color formation, and ovule developmental biology. In addition, the genome sequence will be valuable in studying tree evolution, crop production, and human health, as well as the development of the pomegranate industry.

**References**

Ascacio-Valdes, J.A., Buenrostro-Figueroa, J.J., Aguilera-Carbo, A., Prado-Barragan, A., Rodriguez-Herrera, R. and Aguilar, C.N. (2011) Ellagitannins: biosynthesis, biodegradation and biological properties. *J. Med. Plant Res.* **5**, 4696-4703.

Azuma, A., Ban, Y., Sato, A., Kono, A., Shiraishi, M., Yakushiji, H. and Kobayashi, S. (2015) MYB diplotypes at the color locus affect the ratios of tri/di-hydroxylated and methylated/non-methylated anthocyanins in grape berry skin. *Tree Genet. Genom.* **11**, 31.

Berger, B.A., Kriebel, R., Spalink, D. and Sytsma, K.J. (2016) Divergence times, historical biogeography, and shifts in speciation rates of Myrtales. *Mol. Phylogen. Evol.* **95**, 116-136.

Byng, J.W., Chase, M.W., Christenhusz, M.J.M., Fay, M.F., Judd, W.S., Mabberley, D.J., Sennikov, A.N.*, et al.* (2016) An update of the Angiosperm Phylogeny Group classification for the orders and families of flowering plants: APG IV. *Bot. J. Linn. Soc.* **181**, 1-20.

Colombo, L., Battaglia, R. and Kater, M.M. (2008) *Arabidopsis* ovule development and its evolutionary conservation. *Trends Plant Sci.* **13**, 444-450.

Graham, S.A. (2013) Fossil records in the Lythraceae. *Bot. Rev.* **79**, 48-145.

Graham, S.A. and Graham, A. (2014) Ovary, fruit, and seed morphology of the Lythraceae. *Int. J. Plant Sci.* **175**, 202-240.

Halvorsen, B.L., Holte, K., Myhrstad, M.C.W., Barikmo, I., Hvattum, E., Remberg, S.F., Wold, A.B.*, et al.* (2002) A systematic screening of total antioxidants in dietary plants. *J. Nutr.* **132**, 461-471.

Han, L.L., Yuan, Z.H., Feng, L.J. and Yin, Y.L. (2015) Changes in the composition and contents of pomegranate polyphenols during fruit development. *Acta Hortic.* **1089**, 53-61.

Holland, D., Hatib, K. and Bar-Ya'akov, I. (2009) Pomegranate: botany, horticulture, breeding. In: *Hort. Rev.* (Janick, J. ed) pp. 127-191. Hoboken, NJ, USA: John Wiley & Sons, Inc.

Hu, B., Zhao, J., Lai, B., Qin, Y., Wang, H. and Hu, G. (2016) *LcGST4* is an anthocyanin-related glutathione S-transferase gene in *Litchi chinensis* Sonn. *Plant Cell Rep.* **35**, 831-843.

Johanningsmeier, S.D. and Harris, G.K. (2011) Pomegranate as a functional food and nutraceutical source. *Annu. Rev. Food Sci. Technol.* **2**, 181-201.

Kelley, D.R. and Gasser, C.S. (2009) Ovule development: genetic trends and evolutionary considerations. *Sex. Plant Reprod.* **22**, 229-234.

Maeda, H. and Dudareva, N. (2012) The shikimate pathway and aromatic amino acid biosynthesis in plants. *Annu. Rev. Plant Biol.* **63**, 73-105.

Melgarejo-Sanchez, P., Martinez, J.J., Hernandez, F., Legua, P., Martinez, R. and Melgarejo, P. (2015) The pomegranate tree in the world: new cultivars and uses. *Acta Hortic.* **1089**, 327-332.

Narzary, D., Rana, T.S. and Ranade, S.A. (2010) Genetic diversity in inter-simple sequence repeat profiles across natural populations of Indian pomegranate (*Punica granatum* L.). *Plant Biol.* **12**, 806-813.

Ono, N.N., Britton, M.T., Fass, J.N., Nicolet, C.M., Lin, D. and Tian, L. (2011) Exploring the transcriptome landscape of pomegranate fruit peel for natural product biosynthetic gene and SSR marker discovery. *J. Integr. Plant Biol.* **53**, 800-813.

Ono, N.N., Qin, X., Wilson, A.E., Li, G. and Tian, L. (2016) Two UGT84 family glycosyltransferases catalyze a critical reaction of hydrolyzable tannin biosynthesis in pomegranate (*Punica granatum*). *PLoS One* **11**, e0156319.

Petroni, K. and Tonelli, C. (2011) Recent advances on the regulation of anthocyanin synthesis in reproductive organs. *Plant Sci.* **181**, 219-229.

Pinyopich, A., Ditta, G.S., Savidge, B., Liljegren, S.J., Baumann, E., Wisman, E. and Yanofsky, M.F. (2003) Assessing the redundancy of *MADS-box* genes during carpel and ovule development. *Nature* **424**, 85-88.

Qin, H.N., Graham, S. and Gilbert, M.G. (2007) Lythraceae. In: *Flora of China* (Wu, Z.Y., Raven, P.H. and Hong, D.Y. eds), pp. 274-289. Science Press, Beijing and Missouri GardenPress, Saint Louis.

Teixeira da Silva, J.A., Rana, T.S., Narzary, D., Verma, N., Meshram, D.T. and Ranade, S.A. (2013) Pomegranate biology and biotechnology: a review. *Sci. Hortic.* **160**, 85-107.

Trottier, G., Bostrom, P.J., Lawrentschuk, N. and Fleshner, N.E. (2010) Nutraceuticals and prostate cancer prevention: a current review. *Nat. Rev. Urol.* **7**, 21-30.

Xu, W.J., Dubos, C. and Lepiniec, L. (2015) Transcriptional control of flavonoid biosynthesis by MYB-bHLH-WDR complexes. *Trends Plant Sci.* **20**, 176-185.

Zhao, X.Q., Yuan, Z.H., Feng, L.J. and Fang, Y.M. (2015) Cloning and expression of anthocyanin biosynthetic genes in red and white pomegranate. *J. Plant Res.* **128**, 687-696.


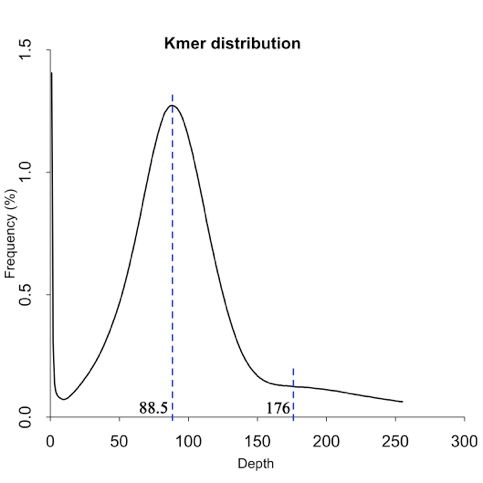


**Figure S1** 17-mer frequency distribution of sequence reads from the library with insert sizes of ~220 bp.


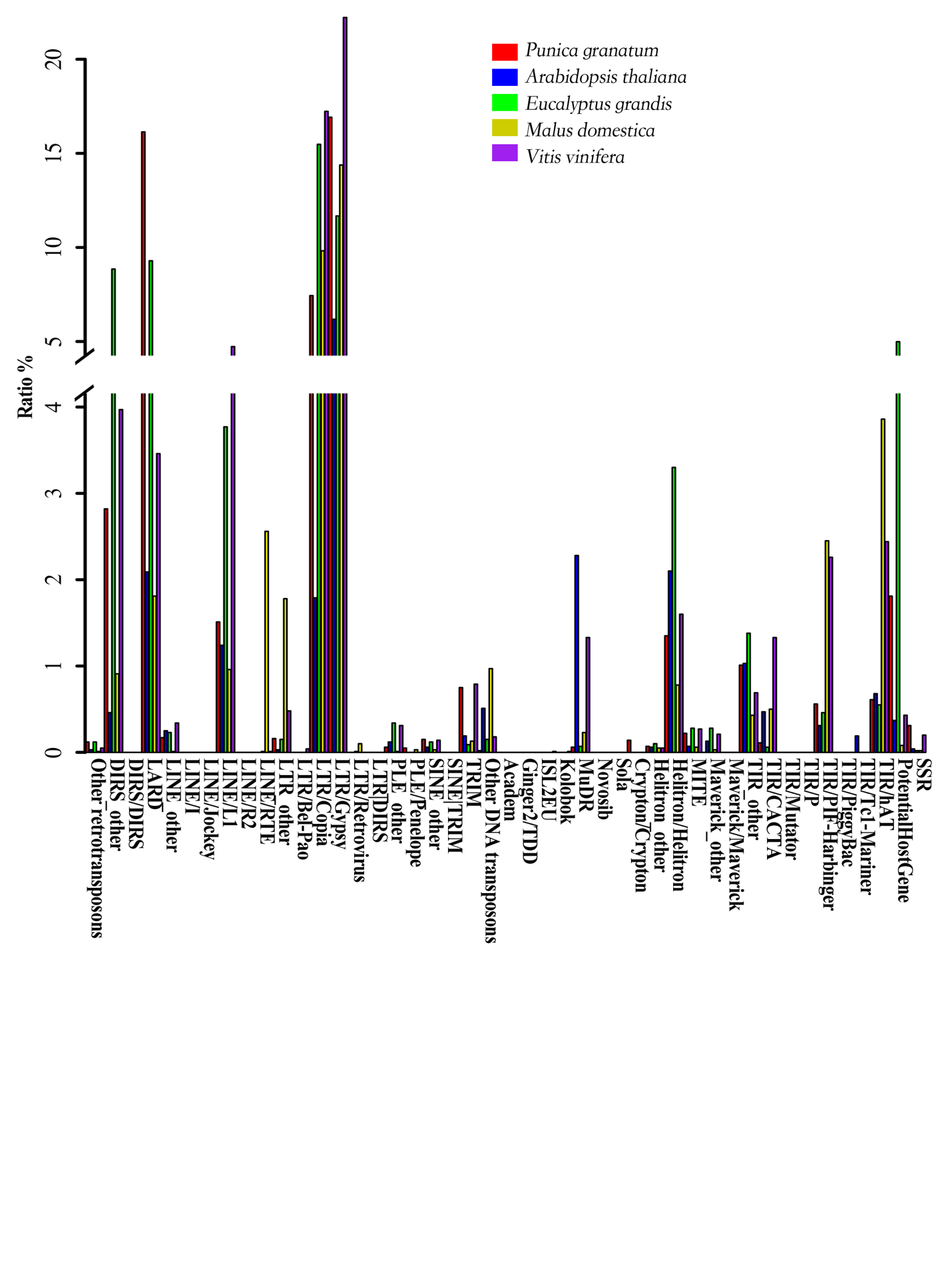


**Figure S2** Comparison of repeat sequences in pomegranate and other plant species.


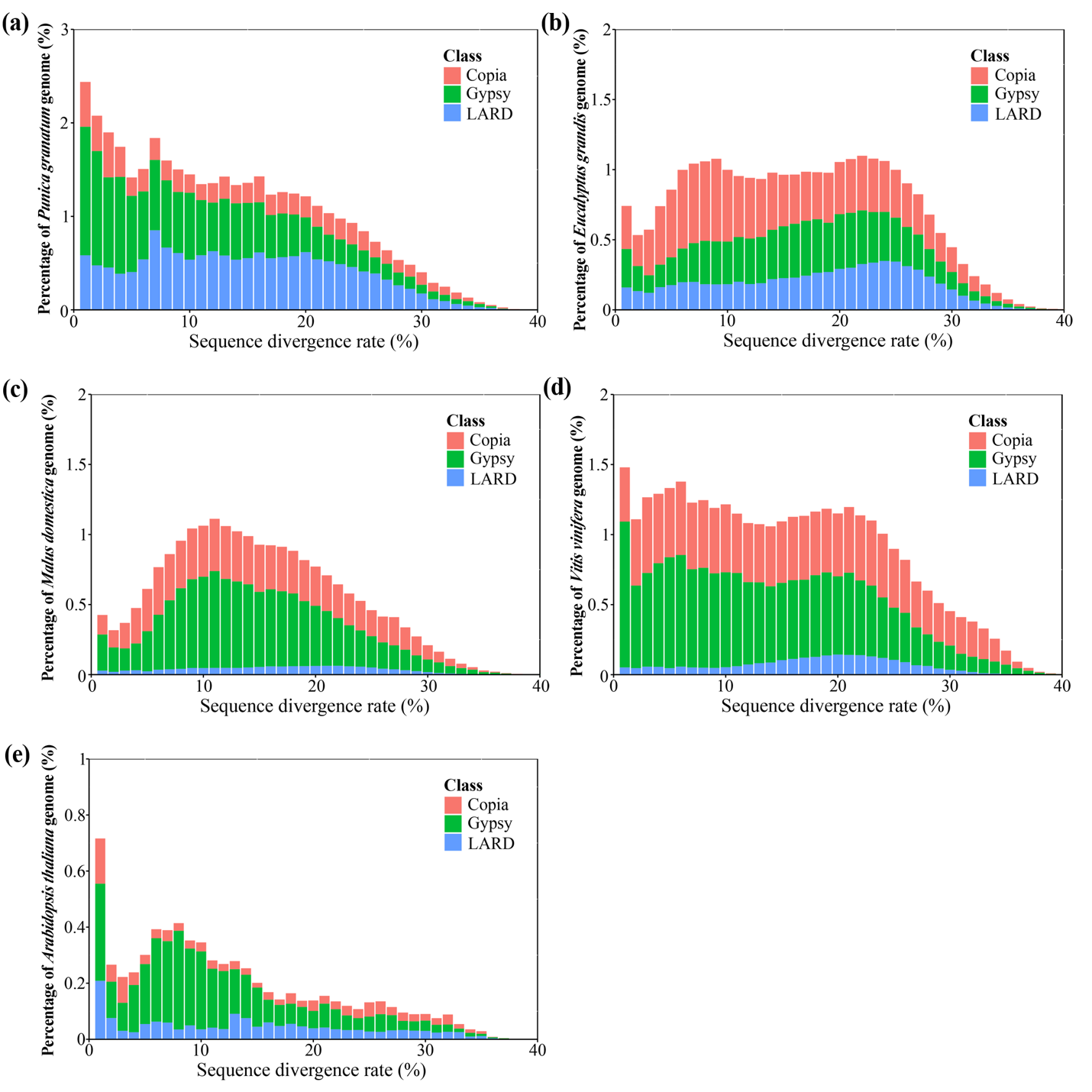


**Figure S3** Distribution of divergence rates of three types of TEs in the genomes of *Punica granatum* (a), *Eucalyptus grandis* (b), *Malus domestica* (c), *Vitis vinifera* (d) and *Arabidopsis thaliana* (e).


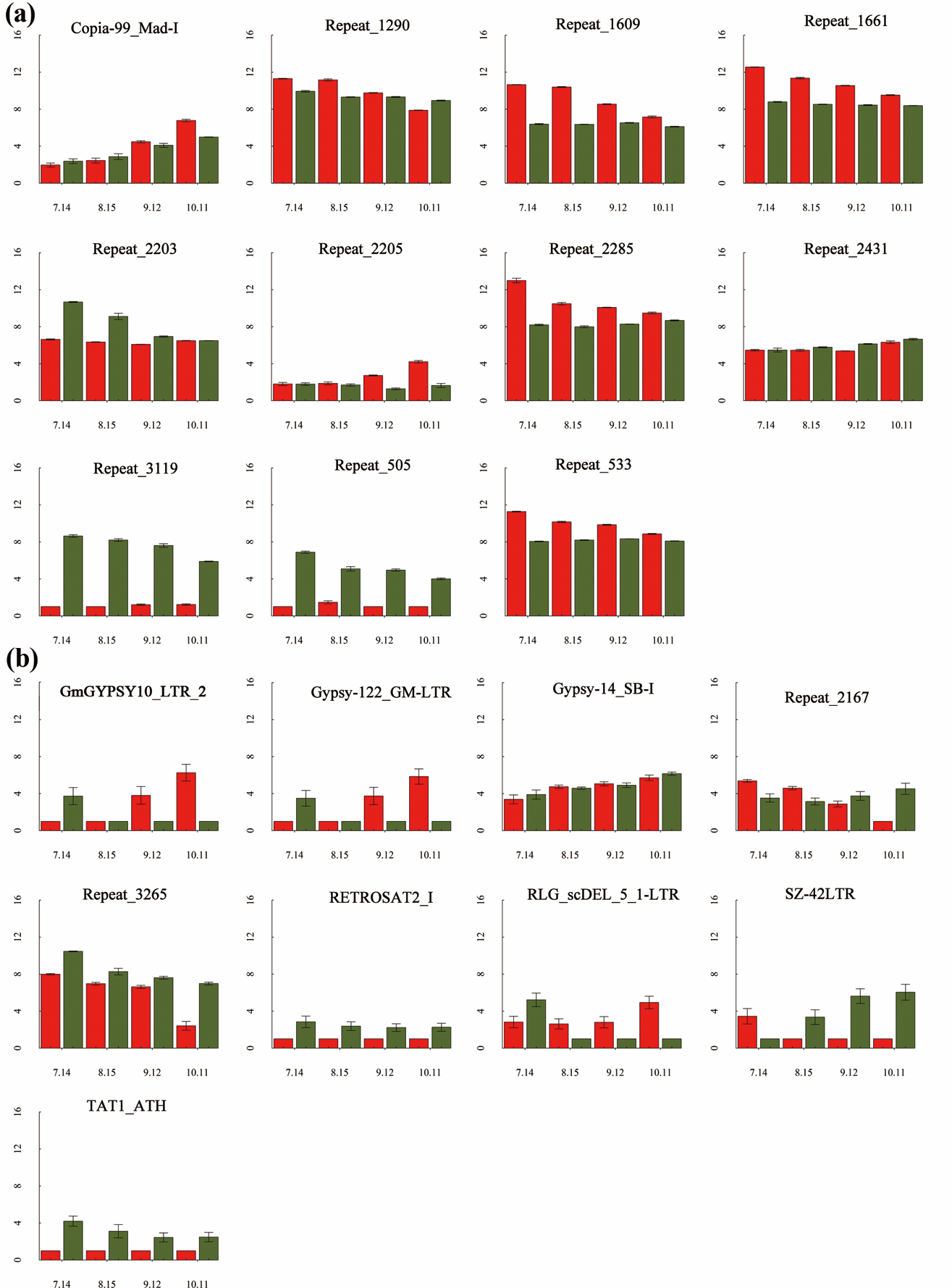


**Figure S4** Expression profiles (TPM; transcripts per million) of a subset of Copia (a) and Gypsy (b) retrotransposons in the peel and aril during pomegranate fruit development.


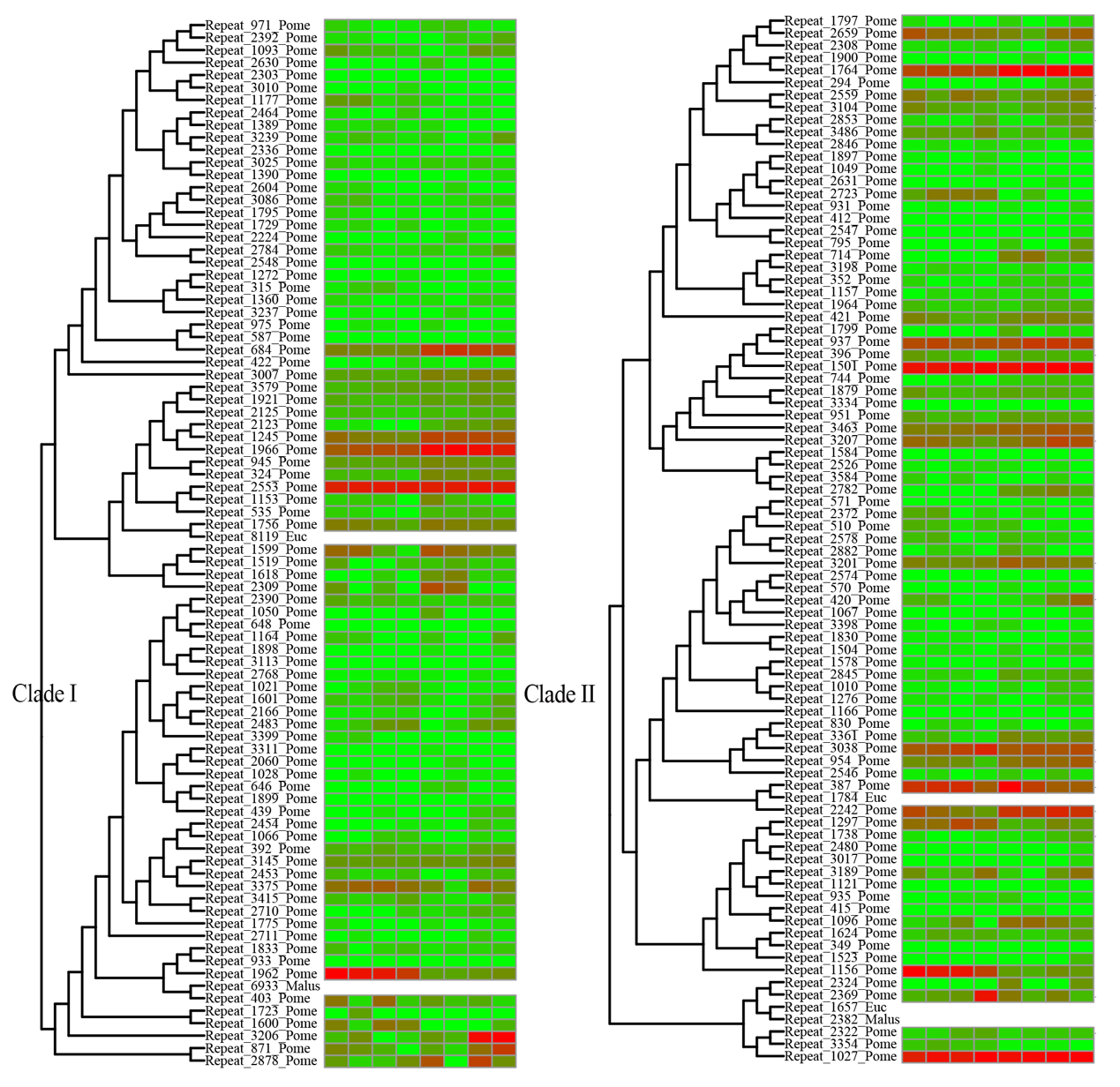


**Figure S5** Expression profiles of large retrotransposon derivatives (LARDs) in the peel and aril during pomegranate fruit development.

**
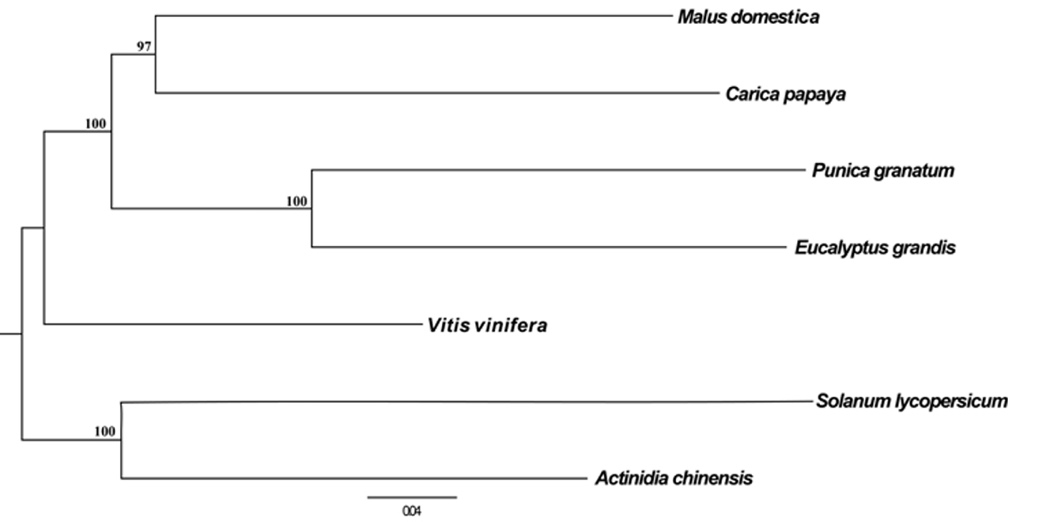
**

**Figure S6** Maximum likelihood (ML) phylogenetic tree of pomegranate and other plant species constructed using single-copy genes.


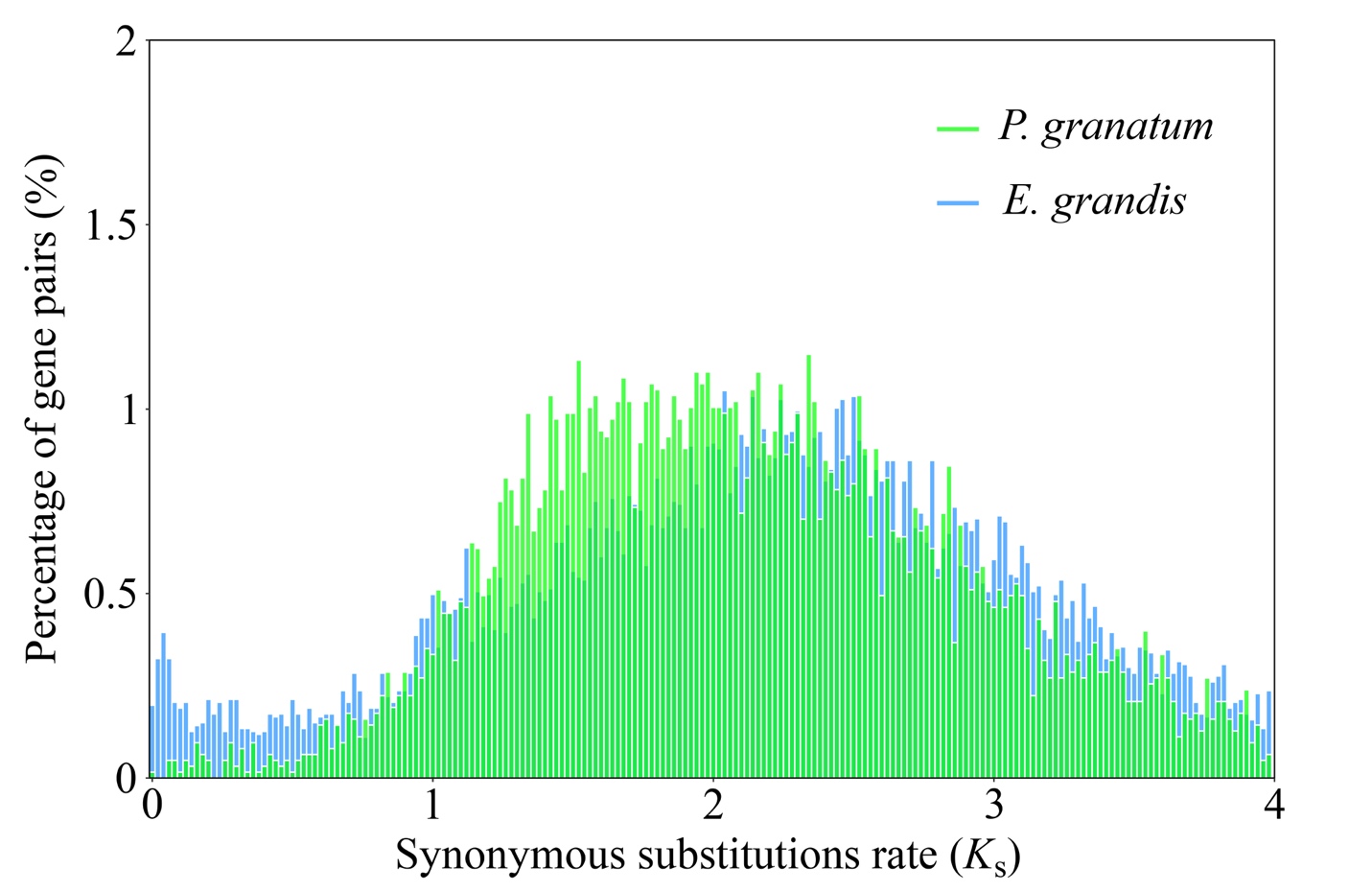


**Figure S7** Distribution of synonymous substitutions rates (*Ks*) of syntenic gene pairs within *Punica granatum* and *Eucalyptus grandis*.


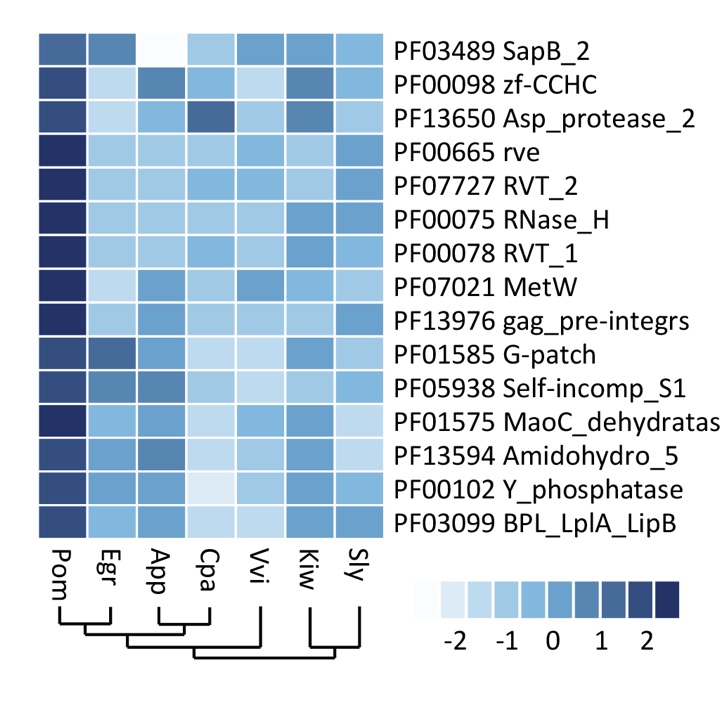


**Figure S8** Expanded gene families in the pomegranate genome. Pom: pomegranate; Egr: Eucalyptus grandis; App: *Arabidopsis* *thaliana*; Cpa: papaya; Vvi: grape; Kiw: kiwifruit; and Sly: tomato.


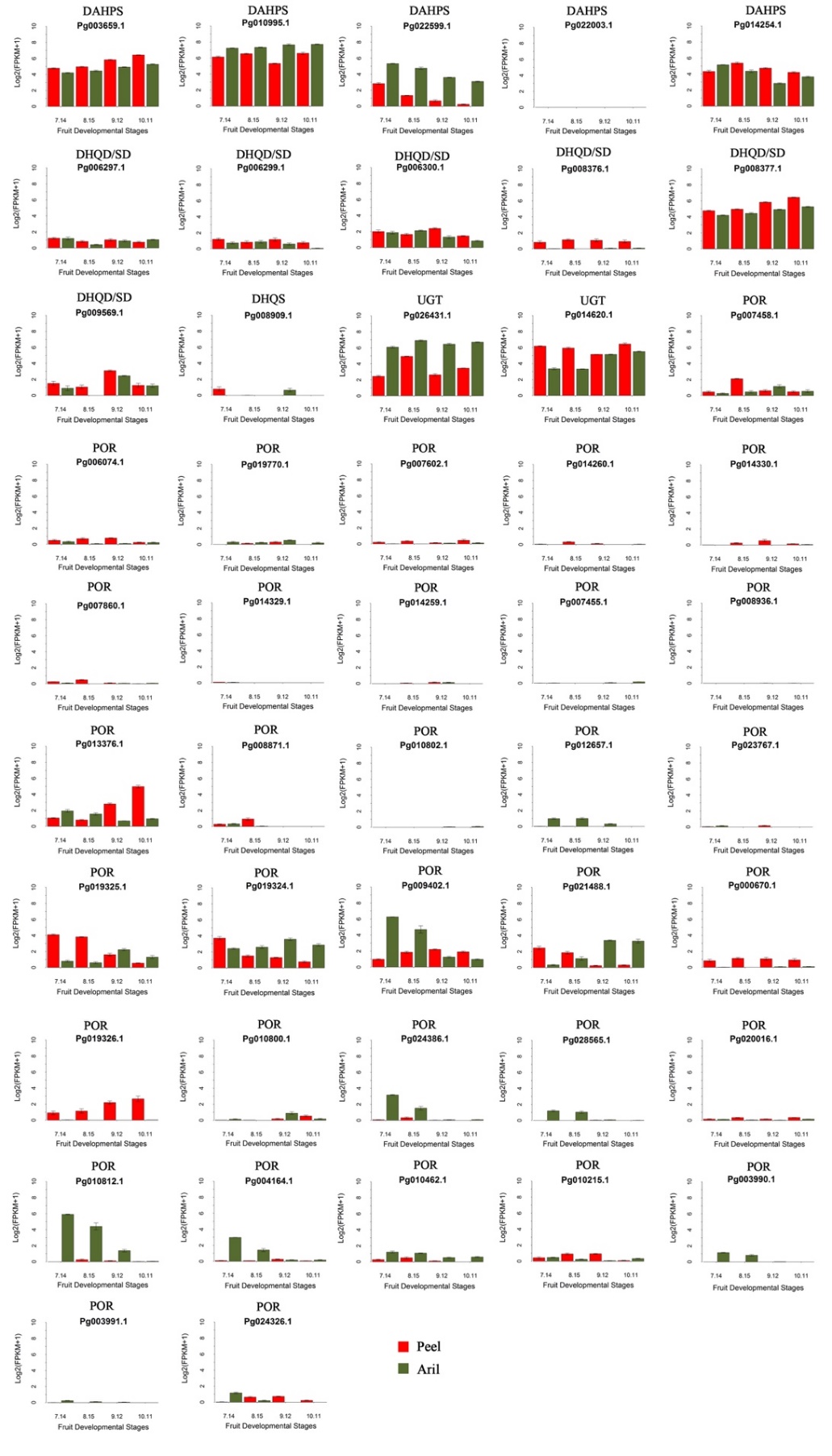


**Figure S9** Expression profiles of the ellagitannin biosynthetic genes in the peel and aril during pomegranate fruit development.


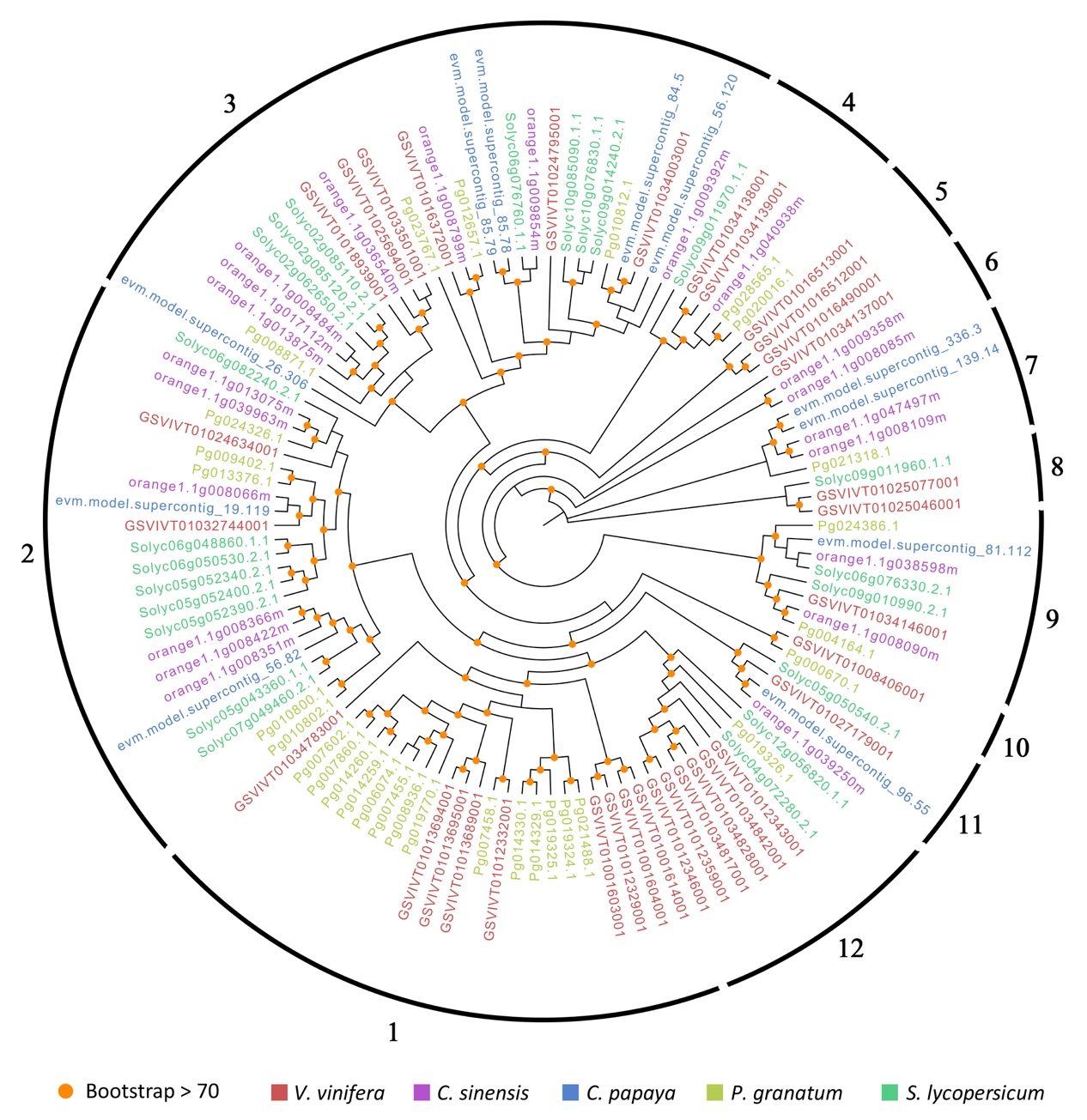


**Figure S10** Phylogenetic tree of pentagalloylglucose oxygen oxidoreductase (POR) genes in pomegranate (*Punica granatum*), grape (*Vitis vinifera*), orange (*Citrus sinensis*), papaya (*Carica papaya*) and tomato (*Solanum lycopersicum*).


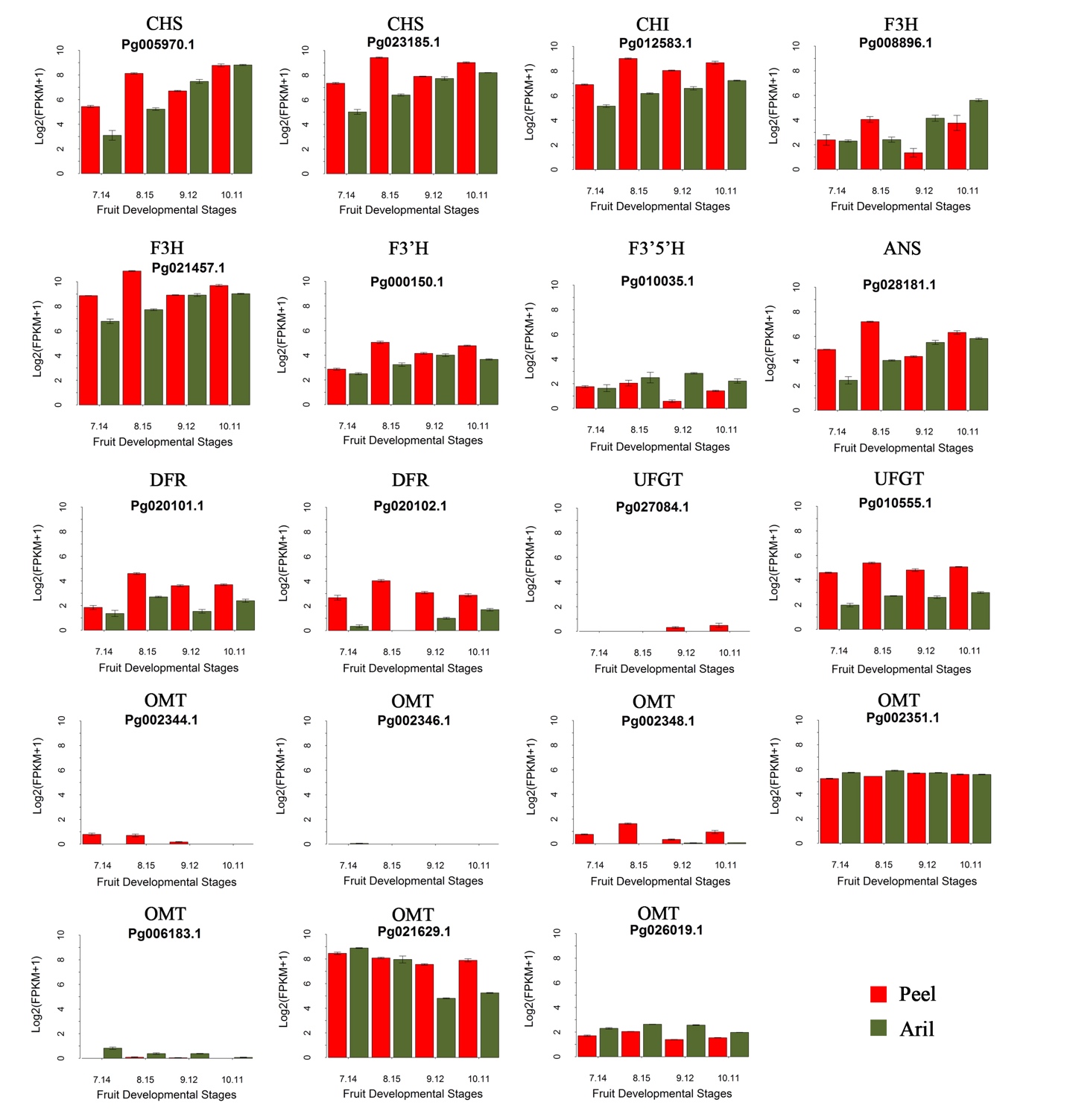


**Figure S11** Expression profiles of the anthocyanin biosynthetic genes in the peel and aril during pomegranate fruit development.


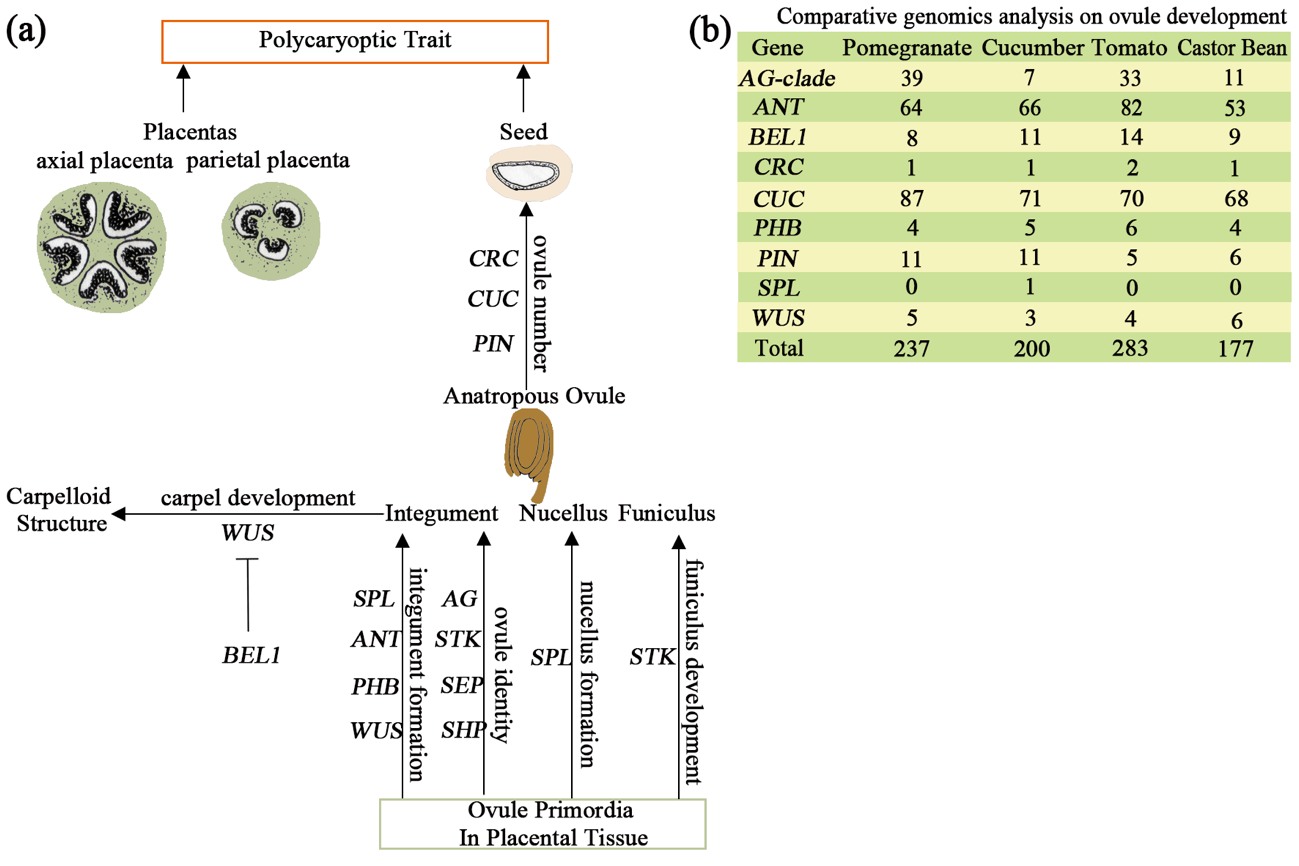


**Figure S12** Regulation of ovule development in pomegranate. (a) Ovule development. Genes involved in the ovule development include those belonging to the MADS-box family, such as AGAMOUS (AG), SEEDSTICK (STK), SEPALLATA (SEP) and SHATTERPROOF (SHP); the HOMEOBOX family, such as WUSCHEL (WUS), PHABULOSA (PHB) and BELL1 (BEL1); the AP2-like family, such as AINTEGUMENTA (ANT); the NOZZLE family, such as SPOROCYTELESS (SPL); the YABBY gene family, such as CRABS CLAW (CRC); and other families, such as CUP-SHAPED COTYLEDONS (CUC) and PIN-FORMED (PIN). (b) Comparative analysis of gene families involved in the ovule development. The AG-clade contains AG, STK, SEP, and SHP genes.

**Table S1** Statistics of the genome sequencing data

| Insert size | Library  Number | Data (Mb) | Depth (X) | Q20 (%) | Q30 (%) |
| --- | --- | --- | --- | --- | --- |
| 220 bp | 1 | 36,681.94 | 109.17 | 96.51 | 89.31 |
| 3 kb | 1 | 3,104.80 | 9.24 | 95.94 | 89.82 |
| 3 kb | 2 | 2,815.19 | 8.38 | 96.13 | 90.15 |
| 4 kb | 1 | 3,037.21 | 9.04 | 97.52 | 92.38 |
| 4 kb | 2 | 3,594.94 | 10.70 | 96.28 | 90.06 |
| 5 kb | 1 | 2,542.14 | 7.57 | 96.02 | 89.78 |
| 5 kb | 2 | 3,404.16 | 10.13 | 96.33 | 90.51 |
| 8 kb | 1 | 4,520.72 | 13.45 | 96.03 | 89.71 |
| 10 kb | 1 | 3,880.41 | 11.55 | 95.98 | 89.61 |
| 15 kb | 1 | 1,775.53 | 5.28 | 96.04 | 89.66 |
| 17 kb | 1 | 1,697.16 | 5.05 | 96.04 | 89.61 |
| Total | 14 | 67,054.21 | 199.57 | -- | -- |

**Table S2** Pomegranate genome size estimated by flow cytometry

| Species | 1C DNA (pg±SD) | Genome Size (Mb) |
| --- | --- | --- |
| Pomegranate | 0.33±0.01 | 322.7±9.8 |
| Rice | 0.45±0.02 | 440.1±19.6 |

Rice (*Oryza sativa* L. spp. *Japonica* var nippobare) was used as the internal reference. The genome size was determined according to the formula: genome size (Mb) = 978 x 1C DNA-value (pg).

**Table S3** Statistics of the final genome assembly

|  | Contig | | Scaffold | |
| --- | --- | --- | --- | --- |
|  | Size (bp) | Number | Size (bp) | Number |
| N50 | 97,003 | 827 | 1,744,793 | 42 |
| N60 | 77,636 | 1,137 | 1,252,576 | 61 |
| N70 | 59,404 | 1,534 | 852,861 | 87 |
| N80 | 42,802 | 2,064 | 556,735 | 126 |
| N90 | 24,287 | 2,890 | 238,441 | 199 |
| Longest | 528,588 | - | 7,666,485 | - |
| Total size | 269,032,625 | - | 274,043,106 | - |
| Total Number (>=100bp) | - | 7,088 | - | 2,117 |
| Total Number (>=1kp) | - | 7,034 | - | 2,117 |

**Table S4** Coverage of expressed sequence tags (ESTs) by the assembled pomegranate genome

| EST  (bp) | Number | Total length (kb) | Bases covered by assembly | Sequences covered by assembly | with >90% sequence in one scaffold | | with >50% sequence in one scaffold | |
| --- | --- | --- | --- | --- | --- | --- | --- | --- |
|  |  |  |  |  | Number | Percent | Number | Percent |
| >0 | 2397 | 1,694 | 94.3% | 99.5% | 2,121 | 88.5% | 2,337 | 97.5% |
| >200 | 2393 | 1,693 | 94.3% | 99.5% | 2,117 | 88.5% | 2,333 | 97.5% |
| >500 | 2168 | 1,603 | 94.9% | 99.9% | 1,991 | 91.8% | 2,130 | 98.3% |

**Table S5** Coverage of unigenes assembled from the RNA-Seq data by the assembled pomegranate genome

| Unigene | Number | Total length (bp) | Bases covered by assembly | Sequences covered by assembly | with >90% sequence in one scaffold | | with >50% sequence in one scaffold | |
| --- | --- | --- | --- | --- | --- | --- | --- | --- |
|  |  |  |  |  | Number | Percent | Number | Percent |
| >0 bp | 70,385 | 55,172,976 | 91.4% | 86.0% | 57,971 | 82.4% | 60,275 | 85.6% |
| >200 bp | 70,385 | 55,172,976 | 91.4% | 86.0% | 57,971 | 82.4% | 60,275 | 85.6% |
| >500 bp | 27,479 | 42,279,362 | 94.7% | 94.3% | 24,595 | 89.5% | 25,812 | 93.9% |

**Table S6** Functional annotation of predicted protein-coding genes

| Database | No. genes annotated | Percentage (%) |
| --- | --- | --- |
| GO | 14,051 | 45.47 |
| KEGG | 5,287 | 17.11 |
| KOG | 15,142 | 49 |
| TrEMBL | 27,148 | 87.85 |
| NR | 27,235 | 88.13 |
| Total annotated | 27,515 | 89.04 |

**Table S7** Non-coding RNAs predicted in the pomegranate genome

| RNA classification | Number | Family |
| --- | --- | --- |
| miRNA | 601 | 270 |
| rRNA | 54 | 3 |
| tRNA | 144 | 41 |

**Table S8** Classification of pomegranate repeat sequences

|  | Type | Number | Length | Percentage of genome (%) |
| --- | --- | --- | --- | --- |
| Retrotransposons | DIRS | 7,266 | 4,399,671 | 1.61 |
|  | LINE | 16,308 | 5,387,936 | 1.97 |
|  | LTR | 1,363 | 594,521 | 0.22 |
|  | LTR/Copia | 30,986 | 16,087,240 | 5.87 |
|  | LTR/Gypsy | 39,879 | 31,658,529 | 11.55 |
|  | PLE\|LARD | 106,206 | 35,611,237 | 12.99 |
|  | SINE | 13,053 | 1,981,942 | 0.72 |
|  | SINE\|TRIM | 1 | 2,202 | 0 |
|  | TRIM | 1,671 | 702,679 | 0.26 |
|  | Unknown | 1,131 | 358,898 | 0.13 |
| DNA transposons | Crypton | 82 | 45,299 | 0.02 |
|  | Helitron | 11,634 | 3,492,839 | 1.27 |
|  | MITE | 16,389 | 4,294,266 | 1.57 |
|  | Maverick | 1,000 | 359,647 | 0.13 |
|  | TIR | 16,598 | 5,001,419 | 1.83 |
|  | Unknown | 3,328 | 356,074 | 0.13 |
|  | PotentialHostGene | 15,286 | 3,827,635 | 1.4 |
| Others | SSR | 8,241 | 930,956 | 0.34 |
| Unknown | Unknown | 76,577 | 25,114,058 | 9.16 |
| Total | Total | 366,999 | 140,207,048 | 51.16 |

**Table S9** Syntenic comparisons between pomegranate, grape and *Eucalyptus grandis* genomes

| Ratio of orthologous regions | grape : pomegranate | | *E. grandis* : pomegranate | |
| --- | --- | --- | --- | --- |
| 1:1 | 5,028 (91.81M) | 3,231 (26.75M) | 23,773 (384.89M) | 20,415 (169.71M) |
| 1:2 | 13,433 (195.88M) | 16,687 (129.68M) | 2,336 (28.83M) | 4,282 (31.05M) |
| 1:3 | 128  (1.96M) | 251  (1.98M) | 28  (0.49M) | 91  (0.56M) |

The number of genes and the total length of genomic regions involved in syntenic blocks are shown.

**Table S10** Number of ellagitannin biosynthetic genes identified in each family in pomegranate and other plant species

| Gene family | *P. granatum* | *E. grandis* | *M. domestica* | *V. vinifera* | *C. sinensis* |
| --- | --- | --- | --- | --- | --- |
| *DAHPS* | 5 | 5 | 9 | 4 | 3 |
| *DHQS* | 1 | 1 | 2 | 1 | 1 |
| *DHQD/SD* | 6 | 5 | 7 | 4 | 3 |
| *UGT* | 2 | 2 | 5 | 1 | 6 |
| *POR* | 34 | 73 | 59 | 75 | 20 |
| Total | 48 | 86 | 82 | 85 | 33 |

**Table S11** Number of anthocyanin biosynthetic genes identified in each family in pomegranate and other plant species

| Gene | *P. granatum* | *E. grandis* | *M. domestica* | *V. vinifera* | *C. sinensis* |
| --- | --- | --- | --- | --- | --- |
| *CHS* | 2 | 4 | 4 | 1 | 4 |
| *CHI* | 1 | 3 | 1 | 1 | 1 |
| *F3H* | 5 | 1 | 1 | 1 | 1 |
| *F3’H* | 2 | 3 | 4 | 1 | 1 |
| *F3’5’H* | 1 | 3 | 4 | 1 | 1 |
| *DFR* | 2 | 1 | 2 | 2 | 1 |
| *ANS/LDOX* | 1 | 1 | 4 | 2 | 1 |
| *UFGT* | 5 | 14 | 4 | 6 | 2 |
| *AOMT* | 7 | 6 | 4 | 7 | 2 |
| Total | 26 | 36 | 28 | 22 | 14 |
